# Clade Age and Species Richness Are Decoupled Across the Eukaryotic Tree of Life

**DOI:** 10.1371/journal.pbio.1001381

**Published:** 2012-08-28

**Authors:** Daniel L. Rabosky, Graham J. Slater, Michael E. Alfaro

**Affiliations:** 1 Department of Integrative Biology and Museum of Vertebrate Zoology, University of California, Berkeley, California, United States of America; 2 Department of Ecology and Evolutionary Biology, University of Michigan, Ann Arbor, Michigan, United States of America; 3 Department of Ecology and Evolutionary Biology, University of California, Los Angeles, California, United States of America; University College London, United Kingdom

## Abstract

Explaining the dramatic variation in species richness across the tree of life remains a key challenge in evolutionary biology. At the largest phylogenetic scales, the extreme heterogeneity in species richness observed among different groups of organisms is almost certainly a function of many complex and interdependent factors. However, the most fundamental expectation in macroevolutionary studies is simply that species richness in extant clades should be correlated with clade age: all things being equal, older clades will have had more time for diversity to accumulate than younger clades. Here, we test the relationship between stem clade age and species richness across 1,397 major clades of multicellular eukaryotes that collectively account for more than 1.2 million described species. We find no evidence that clade age predicts species richness at this scale. We demonstrate that this decoupling of age and richness is unlikely to result from variation in net diversification rates among clades. At the largest phylogenetic scales, contemporary patterns of species richness are inconsistent with unbounded diversity increase through time. These results imply that a fundamentally different interpretative paradigm may be needed in the study of phylogenetic diversity patterns in many groups of organisms.

## Author Summary

Species richness varies by many orders of magnitude across the evolutionary "tree of life." Some groups, like beetles and flowering plants, contain nearly incomprehensible species diversity, but the overwhelming majority of groups contain far fewer species. Many processes presumably contribute to this variation in diversity, but the most general explanatory variable is the evolutionary age of each group: older groups will simply have had more time for diversity to accumulate than younger groups. We tested whether evolutionary age explains differences in species richness by compiling diversity and age estimates for nearly 1,400 groups of multicellular organisms. Surprisingly, we find no evidence that old groups have more species than young groups. This result appears to hold across the entire tree of life, for taxa as diverse as ferns, fungi, and flies. We demonstrate that this pattern is highly unlikely under simple but widely used evolutionary models that allow diversity to increase through time without bounds. Paleontologists have long contended that diversity-dependent processes have regulated species richness through time, and our results suggest that such processes have left a footprint on the living biota that can even be seen without data from the fossil record.

## Introduction

One of the most striking large-scale patterns in biology is the uneven distribution of species richness across the tree of life. Some groups are characterized by nearly incomprehensible species diversity (beetles, grasses), yet many other groups are species-poor (tuataras, ginkgoes). Evolutionary biologists have long been preoccupied with identifying the causal mechanisms underlying these differences in species richness [1]–[3]. These mechanisms include a vast range of biological, historical, and geographic factors. For example, lineage-specific molecular evolutionary traits (e.g., rates of molecular evolution or genome duplication) might be associated with net rates of species diversification [4],[5]. Likewise, species diversification rates might be a function of ecological traits, including those associated with the use of novel resources or defense from natural enemies [6],[7]. The list of factors that have been linked to differential diversification rates is substantial and continues to increase [8]–[11].

The most general explanatory variable of all is clade age [12]: clades vary in age, and this age variation should lead to differences in clade diversity, particularly if all clades have identical net rates of species diversification through time. If clade diversity is generally increasing through time, there is a strong theoretical expectation that species richness should be associated with their age (Figure S1). Even if individual clades are characterized by a “balanced” random walk in diversity, such that speciation and extinction rates are exactly equal, we may still observe a positive relationship between age and richness through time if clade diversity is conditioned on survival to the present day (Figure S1). Stochastic models of clade diversification through time consistently suggest that species richness and clade age should be correlated [13],[14]. These expectations differ from patterns observed for extinct clades [15],[16], presumably because living clades have survived to the present to be observed. The expectation that age and diversity should be correlated does not minimize the importance of evolutionary “key innovations” [7],[17],[18] and other factors as determinants of clade richness. In fact, to the extent that such factors influence net diversification rates, their effects should further accentuate differences in richness attributable to age variation alone.

Surprisingly, previous analyses have reached contrasting conclusions regarding the importance of clade age as a determinant of species richness [12],[13],[19],[20]. For some groups, clade age does not appear to predict species richness, suggesting that clade richness is regulated by diversity-dependence of speciation and extinction rates [14],[21],[22]. Some have suggested that this pattern lacks generality and that that is merely to be expected when clades vary in net diversification rates [20],[23]. The nature of the age-diversity relationship critically influences how we analyze and compare patterns of species richness among clades and between geographic regions. If age and richness truly are decoupled, then species richness in clades should not be modeled as the outcome of a simple time-constant diversification process, as is done in the overwhelming majority of evolutionary and biogeographic studies.

In this study, we evaluate the relationship between clade age and species richness across 1,397 clades of multicellular eukaryotes, including fungi, plants, arthropods, and vertebrates. We explicitly incorporate phylogeny into our analyses to ask the following questions: (i) What is the overall relationship between clade age and species richness across major clades of eukaryotes? (ii) Can simple models of among-clade variation in diversification rates account for the observed relationship between age and richness? (iii) How does the nature of this relationship vary across major subclades of eukaryotes?

## Results

**Figure 1:**
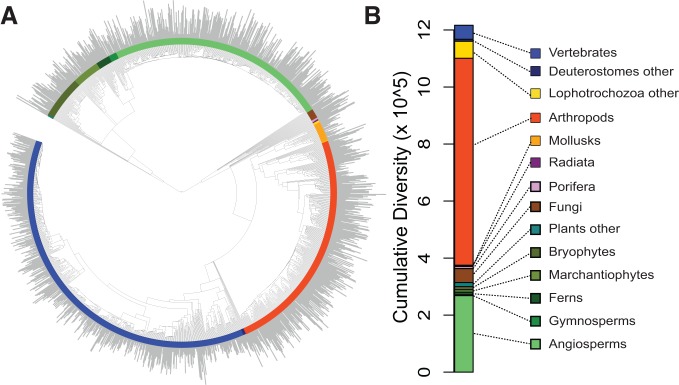
Phylogenetic distribution of species richness across the eukaryotic tree of life. (A) Time-calibrated tree of 1,397 clades of multicellular eukaryotes; length of gray bars indicates relative log-transformed species richness of each group. (B) Total species richness of major groups. Clade colors in (A) correspond to names in (B).

We tested the relationship between clade age and species richness using a recent time-calibrated super-phylogeny [24] that spans virtually the entire tree of life and that contains a record of the phylogenetic relationships and stem clade ages of 1,592 higher taxonomic groups (e.g., families of beetles). We surveyed the literature for data on the extant species richness of all multicellular eukaryotic clades contained within this timetree, including fungi, plants, arthropods, and vertebrates. We obtained richness estimates for a total of 1,397 clades, totaling more than 1.2 million species (Figure 1).

**Figure 2:**
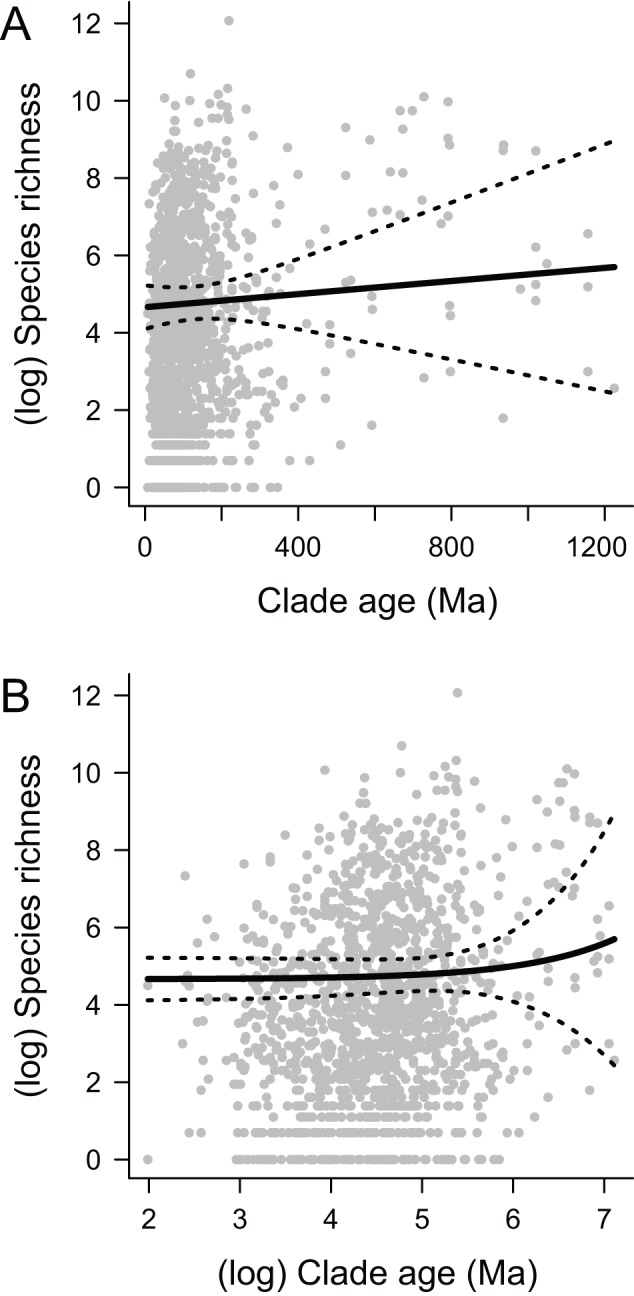
Clade age and species richness are unrelated across 1,397 clades of multicellular eukaryotes. (A) Relationship between log(richness) and clade age (PGLS β = 0.0008, *p* = 0.66). (B) Same relationship as (A), but fitted model is projected onto logarithmic timescale to better visualize the relationship among age and richness for younger clades. The regression coefficient β represents the change in log-transformed diversity per million years.

Using phylogenetic generalized least-squares (PGLS) regression [25], we find no relationship between clade age and log-transformed species richness across the full set of 1,397 major clades of multicellular eukaryotes (Figure 2; *t* = 0.438; *p* = 0.66; *df* = 1395; β = 0.0008, where the regression coefficient β is the change in log-transformed diversity per million years). Use of non-phylogenetic regression models to analyze the age-richness relationship is inappropriate for these data, due to significant phylogenetic signal in clade size across the timetree (variance in independent contrasts test: *p*<10^−20^). We found that high phylogenetic signal in clade size can result in extremely high Type I error rates when the data are analyzed with OLS regression models, even when there is no true relationship between age and diversity (see Materials and Methods; Figure S2). Our results do not break down for younger clades: we found no relationship between age and log-transformed richness for the 307 clades younger than 50 Ma (β = −0.0251; *p* = 0.122; *df* = 305). Similar results were found for other subsets of the data (e.g., subsets of all clades less than 50, 100, 150, 200, and 250 Ma; Table S1; β≤0 for all analyses). Thus, there is no evidence that diversity increases asymptotically with respect to clade age.

**Figure 3:**
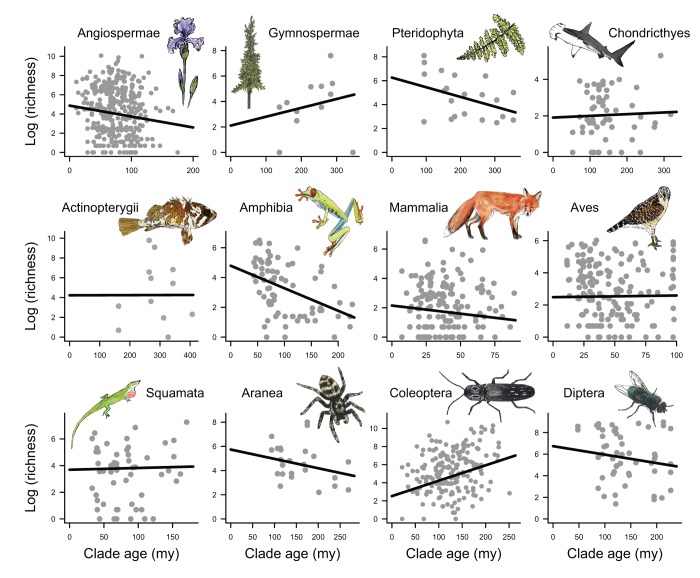
Relationships between age and richness within 12 major taxonomic groups for which dense subclade sampling was available as part of the timetree project [**24**]. Lines represent fitted PGLS relationships between log(richness) and clade age. Beetles show a significant age-diversity relationship (β = 0.017, *p* = 0.004). However, all slopes are less than expected under both relaxed-rate and phylogenetic-rate models of among-clade heterogeneity in net diversification rates (Table 1).

We then examined the relationships between age and richness for the most densely sampled higher taxonomic groups within the timetree (Figure 3). Within this set of 12 major groups (1,133 clades total), only beetles show a significant relationship between age and richness (PGLS β = 0.017, *p* = 0.004). We repeated this analysis across all 352 subtrees within the timetree that contained at least 10 terminal clades and found no evidence that these patterns are simply an artifact of looking at “major” taxonomic groups (Figure S3). Moreover, the significant age-diversity correlation within beetles (Figure 3) is almost entirely attributable to a single subtree containing just 22 terminal clades (Figure S3). Because beetles represent the sole group showing a positive age-diversity correlation, we repeated our analyses on a comprehensive time-calibrated tree of 327 beetle subfamilies from a previous study [26], with the prediction that patterns observed at the family level should hold for more comprehensive subfamily-level sampling. We find no relationship between clade age and species richness at this scale (Figure S4; PGLS β = −0.002, *t* = −0.54; *p* = 0.59; *df* = 325), raising the possibility that the results we observe for beetles are a consequence of the large number of statistical tests we performed. We note that our analyses should have been biased in favor of detecting a significant age-diversity relationship as we did not correct any tests for multiple comparisons.

Substantial variation among clades in net rates of species diversification should weaken the expected relationship between clade age and species richness [14], and previous studies have found that diversification rates show phylogenetic signal across the branches of phylogenetic trees [3],[27],[28]. To address among-clade rate variation, we used the MEDUSA model [3] to estimate the extent of diversification rate variation within each of the 12 major groups shown in Figure 3. MEDUSA analyses strongly supported the presence of multiple rate shifts within each group (Table 1).

**Table 1:** Results of fitting MEDUSA model to 12 higher taxonomic groups with dense subclade sampling.

Taxon	Clades	*N*	AICc-1	AICc-MEDUSA	Shifts	np
Angiosperms	330	268,301	7,914.6	4,743.6	39	119
Gymnosperms	12	2,837	145.8	139.7	1	5
Ferns	21	9,118	538.1	300.5	3	11
Chondrichthyes	57	991	470	426.8	4	14
Actinopterygii	16	18,613	225.4	187.8	1	5
Amphibia	74	6,378	1,253.9	777.7	8	26
Mammalia	149	5,279	1,821.6	1,285	11	35
Aves	163	10,237	2,621.5	1,687.6	15	47
Squamata	53	6,979	896.5	618.1	5	17
Araneae	24	8,776	401.1	332.2	3	11
Coleoptera	183	342,201	3,985.1	2,869.2	17	53
Diptera	51	87,899	1,431.2	906.1	9	29

“Clades” gives the number of subclades within each taxon, and *N* is the total species richness based on our compilation (Table S2). AICc-1 is the Akaike Information Criterion value with finite sample size correction (AICc) for a model with a single set of diversification parameters (speciation, extinction) across the full tree. AICc-MEDUSA is the corresponding AICc value under the best-fit multi-rate model selected by the MEDUSA stepwise procedure. *Shifts* gives number of diversification rate shifts under the best-fit model, and *np* is the corresponding total number of parameters.

The MEDUSA model assumes, but does not test, whether constant-rate diversification processes can account for observed patterns of species richness within higher taxa. To test whether the MEDUSA model of rate variation could result in the age-diversity relationships we report here, we performed *a posteriori* simulations under the fitted MEDUSA parameters and evaluated the model-predicted relationship between clade age and species richness. Performing simulations under the MEDUSA model is challenging, because it requires a stochastic model that can account for the origin of higher taxa as well as for the occurrence of diversification rate shifts on phylogenetic trees. Our implementation assumed a two-state birth-death process, where the units are (i) individual lineages and (ii) higher taxa (see Materials and Methods). We modeled the origin of higher taxa as point occurrence events on the branches of phylogenetic trees; the occurrence of these events can be viewed as analogous to the acquisition of a phenotypic or ecological feature that defines a particular named higher taxon. We further assumed that diversification rate shifts occur within individual lineages under a Poisson process defined by the fitted MEDUSA model. We computed the Spearman correlation between clade age and species richness for each age-diversity dataset generated by the MEDUSA process and compared these distributions to the observed rank-correlations.

**Figure 4:**
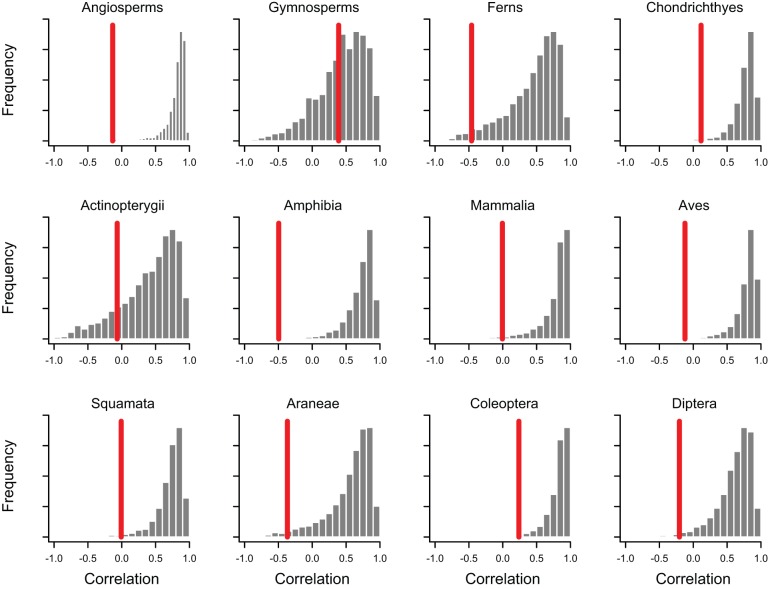
Distributions of rank-order correlations between clade age and species richness predicted under MEDUSA model of rate variation for 12 major taxonomic groups. Vertical red lines show the observed correlation for each group. Observed correlations are significantly lower than the corresponding model-predicted value for 10 of the 12 groups. The high variance of the MEDUSA-predicted distributions for gymnosperms and actinopterygiians is largely explained by the small number of clades (*N* = 12) available for those groups (Figure S5).

Our results indicate that the MEDUSA model of rate variation cannot explain the observed lack of relationship between clade age and species richness (Figure 4). For 10 of the 12 groups, the observed correlation between clade age and species richness is significantly less than the model-predicted correlation (*p*<0.05). Even for beetles, the correlation between age and richness is much lower than expected under the MEDUSA model (*p*<0.002). The two groups for which the MEDUSA model could potentially explain the observed age-diversity correlation (actinopterygiians and gymnosperms) were characterized by the smallest number of subclades (*N* = 12 in each case). The mean age-diversity correlation for each null distribution (Figure 4) is highly correlated with the number of subclades in the dataset (*r* = 0.88; *p*<0.001; Figure S5), suggesting that the effects observed for actinopterygiians and gymnosperms may be manifestations of small sample sizes.

The MEDUSA-based simulations described above are explicitly phylogenetic, in that closely related lineages tend to share common diversification parameters. We also considered a non-phylogenetic model of rate variation whereby each clade diversifies under a constant-rate birth-death process but with individual clade rates drawn from some overall distribution of rates [13],[14]. We implemented this model in a Bayesian framework, assuming that clade rates were drawn from a lognormal distribution [14] but with no phylogenetic signal in the resulting distribution of rates. To test whether this “relaxed rate” model could explain the lack of relationship between age and richness, we conducted posterior predictive simulation by (i) sampling parameters from their joint posterior distributions under the model, (ii) using the sampled parameters to simulate clade species richness, and (iii) using PGLS to evaluate the relationship between clade age and (simulated) species richness. We then computed the standardized effect size (SES) for the observed PGLS slopes to determine whether the observed age-diversity correlation is less than expected if net diversification rates among clades follow a simple lognormal distribution.

As with the MEDUSA simulations (Figure 4), our results reject the hypothesis that among-clade variation in net diversification rates can explain the lack of relationship between age and richness (Table 2). For every combination of subclade and relative extinction rate, the observed slope of the age diversity relationship is lower than the corresponding model-predicted value.

**Table 2:** Age-richness relationships within 12 higher taxonomic groups with dense subclade sampling, compared to expected relationships under a relaxed-rate model of among-clade variation in net diversification rates.

Taxon	Clades	*N*	β (*p*)	SES (ε = 0)	SES (ε = 0.99)
Angiosperms	330	268,301	−0.009 (0.31)	−5.89 (<0.01)	−4.51 (<0.01)
Gymnosperms	12	2,837	0.007 (0.61)	−1.29 (0.10)	−0.38 (0.35)
Ferns	21	9,118	−0.008 (0.41)	−2.44 (0.01)	−1.93 (0.03)
Chondrichthyes	57	991	0.001 (0.82)	−2.77 (<0.01)	−1.83 (0.03)
Actinopterygii	16	18,613	0 (0.99)	−1.01 (0.15)	−0.55 (0.29)
Amphibia	74	6,378	−0.015 (0.10)	−2.88 (<0.01)	−2.34 (0.01)
Mammalia	149	5,279	−0.011 (0.41)	−3.58 (<0.01)	−2.98 (<0.01)
Aves	163	1,0237	0.001 (0.93)	−3.47 (<0.01)	−2.63 (<0.01)
Squamata	53	6,979	0.001 (0.91)	−2.59 (<0.01)	−1.72 (0.04)
Araneae	24	8,776	−0.008 (0.40)	−2.58 (<0.01)	−2.03 (0.02)
Coleoptera	183	342,201	0.017 (<0.01)	−4.27 (<0.01)	−1.92 (0.03)
Diptera	51	87,899	−0.008 (0.40)	−3.35 (<0.01)	−2.65 (<0.01)

“Clades” gives the number of subclades within each taxon, and *N* is the total species richness based on our compilation (Table S2). β gives observed PGLS slope for the relationship between log(richness) and clade age (in millions of years) for each group. Two-tailed *p* values for test of null hypothesis (β = 0) are given in parentheses after slope. SES gives the standardized effect sizes of the observed slope relative to model-predicted values under two relative extinction rates (ε); the corresponding cumulative tail probability is given in parentheses.

## Discussion

Clade age and species richness are decoupled across major clades of multicellular eukaryotes. When considering the full set of 1,397 clades, we found no significant relationship between age and species richness. When the data are partitioned into major subgroups (Figure 3), only beetles are found to have a significant age-diversity relationship. However, a more comprehensive analysis of age-diversity relationships in beetles reveals no relationship between age and richness (Figure S4). We found little evidence for positive age-diversity relationships for individual subtrees containing at least 10 terminal lineages (Figure S3). We found that among-clade variation in net diversification rates is unlikely to explain the lack of relationship between age and richness in any subgroup using two general approaches to model heterogeneity in diversification rates (Figure 4; Table 2). A MEDUSA-type model where diversity in taxonomic groups is produced by rate shifts along a phylogenetic backbone predicts strong positive relationships between clade age and species richness (Figure 4) as do non-phylogenetic models of diversification rate variation (Table 2). Even for beetles, the observed correlation between age and richness is significantly lower than expected under all models of diversification rate heterogeneity.

Although error in the estimates of clade age could theoretically weaken an age-diversity relationship, we consider it unlikely that such error accounts for the patterns we report here. We performed simulations to evaluate the amount of error in clade age that would be required to eliminate a true positive relationship between clade age and species richness (Figure S6). Additional work is needed to fully address this problem, but our results suggest that even extreme error in divergence time estimation is unlikely to eliminate this relationship entirely. These results are consistent with analyses suggesting that inferences about diversification rates from higher taxa are relatively robust to uncertainty in divergence times [29].

Our finding that clade age does not predict species richness challenges a fundamental assumption in most phylogeny-based diversity studies. Previous analyses of limited taxonomic scope have reached different conclusions about the relationship between clade age and diversity [12],[13],[20],[30],[31]. Here, we have demonstrated that (i) the lack of relationship between age and richness is a ubiquitous feature of recognized higher taxa and (ii) this pattern cannot be explained by variation in net diversification rates across the tree of life.

A number of possible mechanisms can account for this general pattern: it may reflect diversity-dependence of speciation and extinction rates [1],[32]–[35]; it may reflect a mixture of expanding and declining diversity trajectories across clades; or it may be an artifact of the way we delimit some clades (but not others) as named higher taxonomic groups (e.g., families). It is also possible that a lack of comparability across clades contributes to the overall lack of relationship between age and richness, and it would be interesting to test whether these results hold at finer phylogenetic scales (e.g., genera within families). Regardless of the underlying causal mechanism, a general decoupling of age and diversity at this scale has profound implications for how we measure and compare diversification and species richness across higher taxa.

### The Pattern as Diversity-Dependence

If diversity-dependent processes regulate species richness within clades [1], then clade age should be a poor predictor of species richness [21],[36]. Clade age will predict species richness only when clades are growing through time. This type of diversity-dependent control is fundamentally related to Simpson's notion of “adaptive zones” [18]: higher taxa, such as the clades we consider in this study, would thus represent monophyletic groups of species that have radiated into a set of related ecological niches. This line of reasoning also implies that diversity dynamics are governed by clade-specific carrying capacities.

Macroevolutionary carrying capacities represent an important component of adaptive radiation [37],[38] and are intrinsically linked to the notion that ecological opportunity influences the tempo and mode of species diversification through time [39]–[41]. We may not understand the ecological mechanisms underlying “carrying capacity” dynamics, but we must still wrestle with substantial neontological and paleontological evidence for their existence. These include patterns of lineage and phenotype diversification as inferred from molecular phylogenies [40],[42]–[44], diversity rebounds after mass extinction [45]–[47], diversity-dependence of speciation and/or extinction rates [33],[48], long periods of diversity-constancy through time [32],[49], and double-wedge patterns of clade turnover through time [50]. Explosive radiations into novel adaptive zones have also been suggested to underlie long-term patterns of phenotypic evolution in a broad range of taxa [51]. In some groups, morphological innovations appear to have promoted shifts in carrying capacities even within geographically restricted radiations [35].

The central challenge in ascribing diversity-dependent causality to the age-diversity relationship in higher taxa is to explain why carrying capacity dynamics would pertain to sets of named higher taxa. The existence of a clade-specific carrying capacity implies that there is something special about named clades themselves, and there is no reason to accept this explanation if higher taxa are effectively random clades with no special meaning. However, higher taxa are clearly not random draws from the tree of life: major clades frequently comprise sets of taxa that are highly distinct in both phenotypic and ecological space (e.g., whales, bats, and carnivores within mammals). In a Simpsonian framework, recognized higher taxa are those clades that have acquired ecological innovations enabling them to radiate in new regions of ecological space, and there is nothing random about our recognizing them as such.

We note that a positive relationship between age and richness need not imply an absence of diversity-dependent regulation of speciation-extinction dynamics. Indeed, positive relationships between stem clade age and richness are expected even under strong diversity-dependence, at least during the initial phase of diversity expansion [36],[52]. However, once clades have reached carrying capacity, age and richness should become decoupled, as has been observed in analyses of several species-level molecular phylogenies [53],[54].

### The Pattern as Declining Diversity

An alternative explanation for the lack of relationship between age and species richness is that the dataset contains clades undergoing both diversity increase and diversity decline. Paleobiologists have long noted that clades in the fossil record tend to wax and wane through time [1],[15],[50],[55]. At least intuitively, it seems reasonable that older clades are more likely to be on the “decline” phase of a diversity trajectory, as has been suggested for snakes [56]. This would provide an immediate explanation for the observed lack of relationship between age and diversity, and would link the patterns described here to the rise and fall of species richness in the fossil record [1],[15].

We find little evidence for a “hump-shaped” relationship between species richness and time (Figures 2–3), one possible pattern that may be consistent with declining diversity scenarios [15],[56]. However, we have only recently begun to explore the mechanisms by which diversity declines might shape age-diversity relationships in extant clades [56]. Recent studies suggest that it may be difficult to detect the signal of diversity declines even with complete species-level molecular phylogenies [57]. Fully addressing the role of diversity declines will presumably require the integration of neontological with paleontological data [58].

### The Pattern as an Artifact

It is possible that the lack of relationship between clade age and richness is an artifact of the non-random manner by which higher taxa are recognized and which has nothing to do with the underlying process of diversity regulation [14]. Clearly, some property of clades causes us to recognize some as cohesive, named units (Aves, Squamata, Actinopterygii); we know very little about the consequences of such taxonomic ranking.

Perhaps the clades we recognize as higher taxa represent a subset of clades that have accumulated exceptional phenotypic distinctiveness relative to other clades. Such clades might, in turn, be those clades that have had lengthy and independent evolutionary histories during which to accumulate sufficient evolutionary change to merit recognition as a distinct higher taxonomic group. One prediction of this model is that named higher taxa would represent crown clades with exceptionally lengthy stem branches. Thus, higher taxa themselves might represent units delimited (albeit indirectly) by a property related to their age, and this could potentially compromise general conclusions about the relationship between clade age and species richness. Likewise, named higher taxa might correspond to clades that have undergone substantial shifts in the tempo and mode of phenotypic evolution [59]; this property itself might be associated with shifts in the dynamics of species diversification. We can at best acknowledge the possibility that the age-diversity relationship might be a statistical artifact attributable to yet-unknown perceptual biases that cause us to name a select subset of the total set of available clades across the tree of life.

### Implications for Diversity Studies

Constant-rate estimators of “net diversification rate,” which assume a sustained increase in species richness through time, remain exceedingly popular for studying the dynamics of diversification from molecular phylogenetic data [3],[20],[60],[61]. This is undoubtedly due in part to the analytical tractability of these methods. Recent methods have been developed for accommodating temporal changes in rates of species diversification on complete species-level phylogenies [53],[62]–[66], but constant-rate estimates remain widely employed in the study of diversification patterns for higher taxonomic levels (but see [13],[14],[56]). At the phylogenetic scales we consider here, constant-rate diversification rate estimates may not be meaningful. This may also be true for the widely used MEDUSA model of rate variation [3], which appears to be incapable of recovering age-diversity relationships consistent with patterns observed in real datasets. If species richness is independent of stem clade age, time-constant models will misleadingly produce rate estimates that are negatively correlated with clade age. Our results suggest that, when age and diversity are not correlated, the significance of rate estimates in macroevolutionary studies should be interpreted with extreme caution since these estimates may offer little insight into the actual underlying processes that regulate species richness within clades [14],[36]. This is true regardless of the underlying causes of the observed age-diversity relationship: even if the absence of an age-diversity relationship is a statistical artifact of the manner by which we recognize higher taxa, our results imply that estimates of diversification rates for higher taxa may have little to do with the factors that influence clade species richness. We are unaware of any theoretical or empirical evidence demonstrating that “constant rate” estimators of net diversification, as applied to stem ages for extant clades, provide any useful insight into evolutionary processes in the absence of a positive relationship between clade age and species richness.

### Conclusions

The relationship between clade age and species richness is fundamental to interpreting the effects of ecological, life-history, geographic, and other factors on clade diversity. A positive relationship between age and richness implies that species richness in clades is controlled by net rates of species proliferation. A decoupling between age and richness implies that other factors exert primary control on richness, or that clade diversity may be declining through time. The notion that species richness in clades can be decoupled from time seems counterintuitive, but is the expected outcome of diversity-dependent regulation of speciation-extinction dynamics. It is possible that species richness across the clades considered here is shaped by a mixture of processes, including diversity-dependence, declining rates, and rate heterogeneity. We are presently unable to determine the relative importance of these and other candidate processes, but integrating other data types (paleontological data; species-level molecular phylogenies) into studies such as this may provide a fruitful avenue for future research. In addition, further research is needed on the nature of higher taxa and the possibility that the results reported here might be a purely statistical consequence of the non-random process by which systematists have designated some clades as higher taxonomic groups. However, we are not presently aware of any non-biological mechanism that can account for this lack of relationship. Our results suggest that large-scale phylogenetic diversity patterns reflect constraints on species richness within clades rather than sustained diversity increases through time.

## Materials and Methods

### Timetree and Species Richness Data

We used a recently published timetree for the tree of life in our analysis [24]. The timetree represents a synthesis of ∼70 time-calibrated, mostly interfamilial studies generated by experts on major taxonomic groups. Although diverse phylogenetic methods were used to generate and time-calibrate these topologies, high congruence in age estimates was observed between the most inclusive timetrees that linked major subsections of the tree of life together and the lower level timetrees contained within each subsection (see Chapter 3 in reference [24]). The combined timetree thus broadly summarizes our current understanding of the timing of major splits across the tree of life and provides a framework for investigating the tempo of diversification of extant lineages. We tabulated data on species richness of each terminal clade represented in the timetree using counts taken from the literature. We preferentially used data from published compendia of species or online checklists that formed parts of ongoing species databasing efforts. These resources were supplemented with richness estimates from other primary literature sources where no checklists were available. Many higher level clades in the timetree were incompletely sampled. In these instances (Table S2), we assigned richness of missing lineages to their closest sister lineage that was present in the time tree, collapsing clades if necessary. This resulted in a total of 1,226,871 species assigned to 1,397 clades.

### Phylogenetic Signal and Species Richness

We conducted simulations to test whether phylogenetic conservatism in clade size alone could generate significant age-richness correlations. Species richness is typically modeled as a geometric random variable, but incorporating covariance among clades due to shared evolutionary history is challenging. We assumed simply that the logarithm of species richness evolved across the phylogeny under a Brownian motion process. Strictly speaking, this is not a valid process-based model for the distribution of species richness across higher level phylogenetic trees. Specifically, this approach assumes that the “backbone structure” of the phylogeny is independent of the process that gives rise to richness at the tips of tree, as species richness is treated as a variable that can simply evolve across a pre-defined tree. This is unlikely to be valid in general, as both the phylogenetic backbone and the tip richness values presumably reflect common dynamic processes of speciation and extinction. However, our objective in these simulations was simply to test whether phylogenetic signal in clade size per se could lead to spurious relationships between clade age and species richness when no such relationship exists in the data, and we note that previous studies have analyzed this relationship in a non-phylogenetic framework [12],[67].

To loosely parameterize our simulations, we first estimated Pagel's lambda [68], which we denote by Λ, for the distribution of log-transformed species richness across the timetree. We found strong support for phylogenetic signal in log-transformed richness (ΔAIC = 372 in favor of model with Λ>0 versus non-phylogenetic model with Λ = 0; maximum likelihood estimate of Λ = 0.724). Using the maximum likelihood estimate of Λ and the corresponding Brownian motion parameters (root state and variance), we simulated 500 datasets under an unconstrained Brownian motion process with the fitted root state and variance parameters. Each simulation thus generated a distribution of log-transformed richness values, with a level of phylogenetic signal (Λ = 0.724) parameterized from the observed data, but with species richness values that are independent of clade age. Significant correlations between clade age and species richness were nonetheless observed in a majority of simulated datasets (Figure S2), despite no relationship between age and richness in the simulation model. This suggests that a simple tendency for closely related clades to be similar in size can lead to a highly misleading perspective on the relationship between age and richness and potentially explains positive age-diversity correlations reported in previous non-phylogenetic analyses [12],[67].

### Posterior Simulations under the MEDUSA Model

The MEDUSA algorithm [3] attempts to identify a mixture of constant-rate birth-death processes that can explain patterns of species richness across higher level phylogenetic trees. We fit the MEDUSA model to the 12 core “higher taxa” with substantial within-group sampling (see Figure 3). It was not feasible to fit a single model to the full dataset of 1,397 clades. Briefly, the algorithm uses a forward stepwise model selection procedure to incrementally add rate-shifts to a phylogenetic tree. The process ends when the addition of a new rate shift fails to improve the log-likelihood of the data beyond a pre-determined AICc (AICc, Akaike Information Criterion with finite sample size correction) threshold. These AICc thresholds for each subtree of *N* taxa were determined using the threshold selection function as implemented in the GEIGER package [69], where the threshold is computed as ΔAICc = A*(N−B)^C^+D. Default values for these parameters in GEIGER are A = −35.94105, B = 6.73726, C = −0.10062, and D = 27.51668. We modified the source code in the original MEDUSA implementation to allow extinction rates to exceed speciation rates, thus enhancing our ability to detect the signal of declining clade diversity through time.

We tested whether the MEDUSA model of rate variation could explain the observed lack of relationship between clade age and species richness by performing *a posteriori* simulations under the fitted models. We developed a simulation model for the MEDUSA process that enabled us to generate a phylogenetic backbone tree as well as higher taxonomic groups and associated species richness values. We assumed a two-state birth-death process, with units of (i) individual lineages and (ii) higher taxa. Our model adds two parameters to the speciation (λ) and extinction (μ) rates of the simple birth-death process. First, we assumed that higher taxa originate from individual lineages at a per-lineage rate Φ. These transitions are irreversible: individual lineages can transition to higher taxa, but the reverse transition is not permitted. Second, we assumed that lineages undergo transitions to new diversification rate classes with rate α.

Each simulation was initiated with *n* = 2 lineages, and simulations were run for a length of time equal to the crown age (*T_c_*) of each major group shown in Figure 3. For each lineage, we sampled the waiting time to the next event from an exponential distribution with parameter β = λ+μ+Φ+α; the identity of the event was then sampled with probability proportionate to the event rate. For example, the probability of a higher taxon formation event would be Φ/β. Upon formation of a higher taxon at time *T_1_*, we assumed that the new taxon inherited the speciation and extinction parameters of the parent lineage; this is consistent with the MEDUSA model formulation, which allows rate shifts only along the internal branches of a phylogenetic tree. Given the remaining interval of time until the present day (*t* = *T_c_*−*T_1_*), we then simulated clade richness (given *λ*, *μ*, and *t*) by sampling an integer-valued random variable from the expected distribution of progeny lineages under the birth-death process [70],[71]. We allowed higher taxa to become extinct before the present. The precise time of origin of a particular higher taxon (*T_1_*) cannot be inferred from the reconstructed phylogenetic trees generated by this simulation procedure; we can only know that the events that define higher taxa occurred at some time after the stem clade age of the group. Thus, phylogenetic trees generated by this algorithm are similar to the higher-level phylogenies analyzed in this and many other studies.

We constrained the per-lineage rate of higher taxon formation to be equal to the rate of speciation at any point in time. This decision was motivated by the observation that these rates must be roughly balanced under the model: for each phylogeny containing *N* higher taxa, we note that the interior “backbone phylogeny” necessarily contains N−1 speciation events (including the root node). Failing to allow approximate equality of these rates can lead to simulated trees consisting entirely of just a few higher taxa (if Φ>λ), or to trees consisting primarily of individual lineages that reached the end of the simulation without forming a higher taxon (if λ>Φ).

Each simulation was initiated by sampling a matched pair of speciation and extinction rates from the set of fitted rate classes inferred under the MEDUSA model. For the diptera, for example, we inferred nine rate shifts under MEDUSA, corresponding to a total of 10 rate classes (including the ancestral rates at the root). When a rate shift event occurred during the simulation, we sampled (with replacement) another matched pair of speciation-extinction rates from the set of fitted MEDUSA values. We set the shift rate equal to the maximum likelihood estimate under a Poisson process model of rate variation. This is obtained by noting simply that the observed number of rate shifts (e.g., nine for diptera) occurred on the internal branches of the phylogeny; an estimate of the event rate is thus given by the number of inferred events divided by the summed internal branch lengths of the phylogeny.

We automatically rejected any simulations that resulted in an exceptionally large or small number of terminals. We set the rejection threshold at 50% and 150% of the observed number of terminals for each dataset; for a dataset with 100 higher taxa, we would thus reject all simulated phylogenies with fewer than 50 or more than 150 terminals at the end of each simulation. We simulated 5,000 phylogenetic trees for each dataset.

### Relaxed Rate Model

As an alternative to the MEDUSA-based simulations described above, we also used a hierarchical Bayes approach to fit a non-phylogenetic “relaxed rate” model of diversification rate variation [14] to each of the 12 core subsets of the data (e.g., angiosperms, beetles, squamate reptiles) with substantial within-group sampling (see Figure 3). Here, we assumed that the net diversification rates for clades within each dataset were drawn from an uncorrelated lognormal distribution. We fit the model under both low (ε = 0) and high (ε = 0.99) relative extinction rates, where ε is the ratio of extinction to speciation rates. For each dataset (e.g., angiosperms), the model has two hyperparameters: the mean and standard deviation of the lognormal distribution of diversification rates. We used Markov Chain Monte Carlo (MCMC) to approximate the posterior distribution of all parameters and hyperparameters.

To assess whether this model could explain the lack of relationship between clade age and species richness, we conducted posterior predictive simulations by simulating species richness values for each clade under the fitted relaxed rate models. Unlike the MEDUSA analyses described above, these simulations treated the phylogenetic backbone tree as fixed; we thus performed phylogenetic GLS analyses on each simulated dataset. For each set of simulations, we computed the standardized effect size for the observed age-diversity relationship as SES = (β_obs_−β_sim_)/σ_sim_, where β_obs_ is the observed PGLS slope, and β_sim_ and σ_sim_ are the expected mean and standard deviations of the slope from posterior predictive simulation. A negative SES value thus indicates negative displacement of the observed value relative to simulations.

## Supporting Information

Figure S1Conditioned birth-death expectation for the relationship between clade age and species richness under four relative extinction rates, defined as the ratio of the extinction rate (μ) to the speciation rate (λ). (A) and (B) display identical information, but species richness in (A) has been log-transformed. Black line, pure-birth process with μ/λ = 0; red line, μ/λ = 0.5; blue line, μ/λ = 0.9; orange line, “balanced process” with μ = λ. Values of μ and λ were chosen in each case to result in 10,000 species at time t = 100. If μ<λ, the relationship between age and log-transformed richness becomes linear as time becomes large. For the “balanced” process with equal speciation and extinction rates (orange), species richness increases linearly with respect to time (B). Note that these results are conditioned on clade survival to the present: if we do not condition on clade survival, log-transformed species richness for extant clades will show a “pure” linear relationship (e.g., black line for pure birth process), provided that μ<λ. In the unconditioned process, species richness will not be correlated with clade age if μ = λ. However, such a process has an expected diversity of N_0_ species and is unlikely to give rise to clades with many hundreds or thousands of species. Expected richness through time curves under the constant rate birth-death process with a single ancestral lineage is given by:
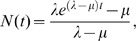
and the conditioned expectation for *N(t)* under the balanced random walk (*μ* = *λ*) is given by *N(t) = 1+λt*.(EPS)

Figure S2 Analysis of relationship between clade age and species richness from non-phylogenetic model (ordinary least-squares regression) when richness values are generated under a model with phylogenetic signal in clade size (see Materials and Methods). Although no relationship between age and richness was input into the simulation model, many simulations yielded datasets with substantial positive and negative age-diversity relationships. Figure shows distribution of *p* values from regressions of log-transformed richness and clade age. Arrow denotes α = 0.05 cutoff. Because there was no relationship between age and richness in the simulation model, simulations with *p* values to the left of this arrow correspond to Type I errors. Phylogenetic signal in clade size alone is thus expected to generate highly significant relationships between age and richness, even when species richness is truly independent of time.
(EPS)

Figure S3 Test of the relationship between clade age and species richness for all possible subtrees with 10 or more descendant clades (352 total). (A) Across the full timetree, a total of 22 subtrees (defined by red circles on nodes) are characterized by significant age-richness relationships. For comparison, subtrees defining sets of clades with significant negative age-richness relationships are shown in blue (11 total). Some “significant” results may simply be due to the large number of statistical tests (e.g., separate PGLS regressions for each of 352 subtrees; significance assessed at α = 0.05, with no correction for multiple comparisons). (B) Significant positive age-richness correlations across the full timetree after removing a single subtree containing 22 beetle clades (arrow). The two remaining significant values (red circles) are also contained within beetles. Most of the effect in (A) can thus be attributed to a single subtree, suggesting a “trickle-down” phenomenon whereby patterns within a single subtree affect analyses at more inclusive nodes/subtrees (e.g., a significant age-richness result for beetles could “trickle down” to drive a significant result across all arthropod clades, simply because beetles are nested within arthropods).
(EPS)

Figure S4 Relationship between clade age and log-transformed species richness for 327 subfamilies of beetles, using phylogeny from Hunt et al. [26]. There is no significant relationship between age and richness for this dataset (PGLS β = −0.002, *t* = −0.54, *p* = 0.59).
(EPS)

Figure S5 Relationship between the mean age-diversity correlation predicted by MEDUSA model of rate variation and the (log-transformed) number of clades in each dataset. The 12 datapoints correspond to the taxonomic subsets (e.g., coleopterans, angiosperms, amphibians) presented in Figure 3.
(EPS)

Figure S6 Effect of error in the estimation of clade age on a true positive relationship between clade age and species richness. We took the observed set of angiosperm clade ages as fixed (*N* = 330 clades) and simulated species richness on that set of ages assuming a constant-rate birth death process for the entire angiosperm radiation. Then, holding these richness values constant, we introduced error into the clade ages. We then computed the correlation between species richness and the “error-modified” vectors of clade ages. A single simulation thus entailed (i) drawing species richness given the observed ages, (ii) introducing error into those ages, and (iii) analyzing the relationship between these modified ages and richness. We assumed that error in clade age estimates followed a normal distribution centered at 0 with a standard deviation equal to δT, where T is the age of the clade and δ is an error parameter taking values of 0.1, 0.2, 0.3, 0.4, 0.5, and 0.6. Thus, error is a linear function of time, and older clades show considerably more uncertainty in clade age than younger clades. The top panel shows the 0.95 percentiles of the distribution of age-diversity correlations under progressively increasing δ. In the bottom panel, we illustrate the amount of uncertainty in clade age implied by a value of δ = 0.6. The black line shows the rank-ordered set of observed angiosperm clade ages; the vertical gray lines denote the corresponding 95% confidence intervals in clade age under this error model. A value of δ = 0.6 can thus result in enormous confidence intervals for some old clades. For example, a clade of age 100 my would have a 95% confidence interval on clade age ranging from 0 to 217.6. Despite this error in clade age, the corresponding age-diversity relationship retains considerable signal of the underlying age-diversity relationship (top panel, δ = 0.6). 1,000 simulations were conducted per value of δ. Speciation and extinction rates for simulations used the observed maximum likelihood estimates for the full angiosperm radiation (λ = 0.71, μ = 0.64). If the error term resulted in a clade age of less than 0, we resampled values until the resulting age was greater than zero.
(EPS)

Table S1 Relationship between stem clade age and species richness for subsets of the data containing young clades only. The full dataset was pruned to contain only those clades younger than a given “truncation age,” and the full PGLS analysis was repeated on each subset. Thus, the analysis for “truncation age = 50” corresponds to the subset of clades younger than 50 Ma (*n* = 307). There was no relationship between age and log-transformed richness for any subset.
(DOC)

Table S2 Richness values for clades represented in the timetree and their associated sources.
(DOC)

## Abbreviations

AIC: Akaike Information Criterion
MCMC: Markov Chain Monte Carlo
MEDUSA: Modeling Evolutionary Diversification Using Stepwise AIC
PGLS: phylogenetic generalized least squares
SES: standardized effect size
