# Supplementary material for: Clade Age and Species Richness Are Decoupled Across the Eukaryotic Tree of Life
Source: PLoS Biol. 2012 Aug 28;10(8):e1001381. doi: 10.1371/journal.pbio.1001381 (PMC3433737; doi:10.1371/journal.pbio.1001381)
Supplement: Table S1 — Relationship between stem clade age and species richness for subsets of the data containing young clades only. The full dataset was pruned to contain only those clades younger than a given “truncation age,” and the full PGLS analysis was repeated on each subset. Thus, the analysis for “truncation age = 50” corresponds to the subset of clades younger than 50 Ma (n = 307). There was no relationship between age and log-transformed richness for any subset. (DOC) [file pbio.1001381.s007.doc]

Table S1. Relationship between stem clade age and species richness for subsets of the data containing young clades only. The full dataset was pruned to contain only those clades younger than a given “truncation age”, and the full PGLS analysis was repeated on each subset. Thus, the analysis for “truncation age = 50” corresponds to the subset of clades younger than 50 Ma (n = 307). There was no relationship between age and log-transformed richness for any subset.

| Truncation age (Ma) | PGLS Slope | Number of clades | p |
| --- | --- | --- | --- |
| 50 | -0.025 | 307 | 0.123 |
| 100 | -0.005 | 764 | 0.464 |
| 150 | -0.007 | 1082 | 0.097 |
| 200 | -0.002 | 1218 | 0.59 |
| 250 | -0.001 | 1281 | 0.81 |
