# Supplementary material for: Clade Age and Species Richness Are Decoupled Across the Eukaryotic Tree of Life
Source: PLoS Biol. 2012 Aug 28;10(8):e1001381. doi: 10.1371/journal.pbio.1001381 (PMC3433737; doi:10.1371/journal.pbio.1001381)
Supplement: Table S2 — Richness values for clades represented in the timetree and their associated sources. (DOC) [file pbio.1001381.s008.doc]

**Table S2.** Richness values for clades represented in the timetree, and their associated sources.

| Taxon | Richness | Ref | Notes |
| --- | --- | --- | --- |
| Bangiales | 6081 | 1 |  |
| Cyanidiales | 6 | 2 |  |
| Glaucopyta | 13 | 3 |  |
| Chlorophyta | 7000 | 4 |  |
| Lycopsida | 1230 | 5 |  |
| Anthocerotophyta | 100 | 5 |  |
| Plagiotheciaceae | 52 | 6 |  |
| Meteroiaceae | 207 | 6 |  |
| Fontinalaceae | 27 | 6 |  |
| Cryphaeaceae | 93 | 6 |  |
| Pterobryaceae | 168 | 6 |  |
| Thuidiaceae | 139 | 6 |  |
| Entodontaceae | 135 | 6 |  |
| Hypnaceae | 679 | 6 |  |
| Brachytheciaceae | 569 | 6 |  |
| Hylocomiaceae | 31 | 6 |  |
| Leucodontaceae | 51 | 6 |  |
| Neckeraceae | 225 | 6 |  |
| Stereophyllaceae | 51 | 6 |  |
| Fabroniaceae | 87 | 6 |  |
| Lepyrodontaceae | 8 | 6 |  |
| Catagoniaceae | 4 | 6 |  |
| Leucomiaceae | 12 | 6 |  |
| Pilotrichaceae | 569 | 6 |  |
| Hookeriaceae | 57 | 6 |  |
| Trachylomataceae | 8 | 6 |  |
| Rutenbergiaceae | 7 | 6 |  |
| Hypopterygiaceae | 73 | 6 |  |
| Ptychomniaceae | 42 | 6 |  |
| Aulacomniaceae | 6 | 6 |  |
| Pterobryellaceae | 6 | 6 |  |
| Braithwaiteaceae | 1 | 6 |  |
| Racopilaceae | 61 | 6 |  |
| Hypnodendraceae | 27 | 6 |  |
| Orthodontiaceae | 14 | 6 |  |
| Rhizogoniaceae | 34 | 6 |  |
| Hedwigiaceae | 237 | 6 |  |
| Bartramiaceae | 387 | 6 |  |
| Mniaceae | 88 | 6 |  |
| Bryaceae | 609 | 6 |  |
| Orthotrichaceae | 841 | 6 |  |
| Splachnaceae | 72 | 6 |  |
| Meesiaceae | 19 | 6 |  |
| Timmiaceae | 5 | 6 |  |
| Scouleriaceae | 4 | 6 |  |
| Pottiaceae | 1100 | 6 |  |
| Ditrichaceae | 900 | 6 |  |
| Fissidentaceae | 447 | 6 |  |
| Dicranaceae | 983 | 6 |  |
| Schistostegaceae | 1 | 6 |  |
| Seligeriaceae | 81 | 6 |  |
| Ptychomitriaceae | 179 | 6 |  |
| Grimmiaceae | 276 | 6 |  |
| Funariaceae | 255 | 6 |  |
| Encalpytaceae | 35 | 6 |  |
| Diphysciaceae | 21 | 6 |  |
| Buxbaumiaceae | 12 | 6 |  |
| Tetraphidaceae | 5 | 6 |  |
| Polytrichaceae | 218 | 6 |  |
| Oedipodiaceae | 215 | 6 |  |
| Andreaeaceae | 95 | 6 |  |
| Andreaeobryaceae | 1 | 6 |  |
| Takakiaceae | 2 | 6 |  |
| Sphagnaceae | 288 | 6 |  |
| Calypogeiaceae | 119 | 7 |  |
| Arnelliaceae | 29 | 7 |  |
| Gymnomitriaceae | 56 | 7 |  |
| Jungermanniaceae_2 | 154 | 7 |  |
| Balantiopsidaceae | 96 | 7 |  |
| Acrobolbaceae | 69 | 7 |  |
| Myliaceae | 6 | 7 |  |
| Scapaniaceae | 454 | 7 |  |
| Cephaloziellaceae | 213 | 7 |  |
| Cephaloziaceae | 193 | 7 |  |
| Adelanthaceae | 18 | 7 |  |
| Lophocoleaceae | 503 | 7 |  |
| Plagiochilaceae | 1000 | 7 |  |
| Herbertaceae | 66 | 7 |  |
| Lepicoleaceae | 18 | 7 |  |
| Lepidoziaceae | 847 | 7 |  |
| Trichocoleaceae | 51 | 7 |  |
| Pseudolepicoleaceae | 42 | 7 |  |
| Schistochilaceae | 105 | 7 |  |
| Lejeuneaceae | 2237 | 7 |  |
| Jubulaceae | 3 | 7 |  |
| Frullaniaceae | 827 | 7 |  |
| Radulaceae | 245 | 7 |  |
| Porellaceae | 171 | 7 |  |
| Lepidolaenaceae | 18 | 7 |  |
| Goebeliellaceae | 1 | 7 |  |
| Ptilidiaceae | 11 | 7 |  |
| Makinoaceae | 1 | 7 |  |
| Aneuraceae | 454 | 7 |  |
| Metzgeriaceae | 121 | 7 |  |
| Pleuroziaceae | 13 | 7 |  |
| Pallaviciniaceae | 81 | 7 |  |
| Hymenophytaceae | 2 | 7 |  |
| Fossombroniaceae | 95 | 7 |  |
| Pelliaceae | 19 | 7 |  |
| Marchantiales | 312 | 7 |  |
| Sphaerocarpales | 36 | 7 |  |
| Blasiales | 11 | 7 |  |
| Treubiales | 16 | 7 |  |
| Haplomitriales | 14 | 7 |  |
| Eupolypods_II_2 | 1855 | 8 |  |
| Eupolypods_II_1 | 700 | 8 |  |
| Eupolypods_I_2 | 3292 | 8 |  |
| Eupolypods_I_1 | 13 | 9 |  |
| Pteridaceae | 950 | 8 |  |
| Dennstaedtiaceae | 170 | 8 |  |
| Lindsaeoids1 | 212 | 8 |  |
| Tree_ferns_2 | 20 | 8 |  |
| Tree_ferns_1 | 643 | 8 |  |
| Salviniaceae | 16 | 8 |  |
| Marsileaceae | 75 | 8 |  |
| Schizaeoids_2 | 130 | 8 |  |
| Schizaeoids_1 | 25 | 8 |  |
| Gleichenioids_2 | 15 | 8 |  |
| Gleichenioids_1 | 125 | 8 |  |
| Hymenophyllaceae | 600 | 8 |  |
| Osmundaceae | 20 | 8 |  |
| Marattiaceae | 150 | 8 |  |
| Equisetaceae | 15 | 8 |  |
| Psilotaceae | 12 | 8 |  |
| Ophioglossaceae | 80 | 8 |  |
| Podocarpaceae | 180 | 10 |  |
| Araucariaceae | 35 | 10 |  |
| Cupressaceae | 173 | 10 |  |
| Cephalotaxaceae | 12 | 10 |  |
| Taxaceae | 25 | 10 |  |
| Welwitschiaceae | 1 | 10 |  |
| Gnetaceae | 35 | 10 |  |
| Ephedraceae | 50 | 10 |  |
| Pinaceae | 225 | 10 |  |
| Ginkgoaceae | 1 | 10 |  |
| Cycadaceae | 97 | 10 |  |
| Zamiaceae | 2003 | 10 |  |
| Amborellales | 1 | 12 |  |
| Nymphaeales | 74 | 12 |  |
| Austrobaileyales | 100 | 12 |  |
| Chloranthales | 75 | 12 |  |
| Annonaceae | 2220 | 12 |  |
| Eupomatiaceae | 3 | 12 |  |
| Degeneriaceae | 2 | 12 |  |
| Himantandraceae | 2 | 12 |  |
| Magnoliaceae | 227 | 12 |  |
| Myristicaceae | 475 | 12 |  |
| Winteraceae | 90 | 12 |  |
| Canellaceae | 13 | 12 |  |
| Calycanthaceae | 11 | 12 |  |
| Lauraceae | 2500 | 12 |  |
| Gomortegaceae | 1 | 12 |  |
| Atherospermataceae | 16 | 12 |  |
| Monimiaceae | 200 | 12 |  |
| Hernandiaceae | 55 | 12 |  |
| Saururaceae | 6 | 12 |  |
| Piperaceae | 3615 | 12 |  |
| Aristolochiaceae | 480 | 12 |  |
| Ceratophyllales | 2 | 12 |  |
| Vitaceae | 850 | 12 |  |
| Salicaceae | 1010 | 12 |  |
| Lacistemataceae | 14 | 12 |  |
| Violaceae | 800 | 12 |  |
| Passifloraceae | 705 | 12 |  |
| Goupiaceae | 800 | 12 |  |
| Achariaceae | 145 | 12 |  |
| Rhizophoraceae | 149 | 12 |  |
| Erythroxylaceae | 240 | 12 |  |
| Caryocaraceae | 21 | 12 |  |
| Irvingiaceae | 10 | 12 |  |
| Medusagynaceae | 1 | 12 |  |
| Ochnaceae | 495 | 12 |  |
| Quiinaceae | 55 | 12 |  |
| Putranjivaceae | 210 | 12 |  |
| Humiriaceae | 50 | 12 |  |
| Malpighiaceae | 1250 | 12 |  |
| Hypericaceae | 560 | 12 |  |
| Podostemaceae | 270 | 12 |  |
| Clusiaceae | 595 | 12 |  |
| Picrodendraceae | 80 | 12 |  |
| Balanopaceae | 9 | 12 |  |
| Chrysobalanaceae | 460 | 12 |  |
| Dichapetalaceae | 165 | 12 |  |
| Trigoniaceae | 28 | 12 |  |
| Euphorbiaceae | 5735 | 12 |  |
| Linaceae | 300 | 12 |  |
| Oxalidaceae | 770 | 12 |  |
| Cunoniaceae | 280 | 12 |  |
| Elaeocarpaceae | 605 | 12 |  |
| Huaceae | 3 | 12 |  |
| Celastraceae | 1350 | 12 |  |
| Parnassiaceae | 51 | 12 |  |
| Fabaceae | 19400 | 12 |  |
| Polygalaceae | 940 | 12 |  |
| Surianaceae | 8 | 12 |  |
| Betulaceae | 146 | 12 | includes sister taxon Ticodendraceae [ = 1sp] |
| Casuarinaceae | 95 | 12 |  |
| Juglandaceae | 51 | 12 | includes sister taxon Rhoipteleaceae [ = 1sp] |
| Myricaceae | 57 | 12 |  |
| Fagaceae | 670 | 12 |  |
| Begoniaceae | 1401 | 12 |  |
| Tetramelaceae | 2 | 12 |  |
| Datiscaceae | 2 | 12 |  |
| Coriariaceae | 5 | 12 |  |
| Corynocarpaceae | 6 | 12 |  |
| Cucurbitaceae | 845 | 12 |  |
| Barbeyaceae | 1 | 12 |  |
| Elaeagnaceae | 45 | 12 |  |
| Rhamnaceae | 925 | 12 |  |
| Urticaceae | 2625 | 12 |  |
| Celtidaceae | 170 | 12 | Cannabaceae in [12] |
| Moraceae | 1125 | 12 |  |
| Ulmaceae | 35 | 12 |  |
| Rosaceae | 2520 | 12 |  |
| Zygophyllaceae | 18 | 12 |  |
| Krameriaceae | 285 | 12 |  |
| Onagraceae | 656 | 12 |  |
| Lythraceae | 620 | 12 |  |
| Combretaceae | 500 | 12 |  |
| Myrtaceae | 4620 | 12 |  |
| Vochysiaceae | 190 | 12 |  |
| Melastomataceae | 5046 | 12 | includes sister taxa Alzateaceae [ = 2sp], Penaeaceae [= 29sp], Crypteroniaceae [= 10sp] |
| Sapindaceae | 1580 | 12 |  |
| Simaroubaceae | 95 | 12 |  |
| Meliaceae | 621 | 12 |  |
| Rutaceae | 1815 | 12 |  |
| Burseraceae | 550 | 12 |  |
| Anacardiaceae | 680 | 12 |  |
| Dipterocarpaceae | 500 | 12 |  |
| Sarcolaenaceae | 60 | 12 |  |
| Cistaceae | 175 | 12 |  |
| Muntingiaceae | 13 | 12 |  |
| Thymelaeaceae | 891 | 12 |  |
| Bixaceae | 15 | 12 |  |
| Malvaceae | 4225 | 12 |  |
| Neuradaceae | 10 | 12 |  |
| Akaniaceae | 2 | 12 |  |
| Tropaeolaceae | 95 | 12 |  |
| Bataceae | 13 | 12 | includes Salvadoraceae [= 11sp] |
| Koeberliniaceae | 1 | 12 |  |
| Brassicaceae | 4410 | 12 | includes Cleomaceae [= 300sp], Capparaceae [= 400sp] |
| Resedaceae | 93 | 12 | includes Gyrostemonaceae [= 18sp] |
| Limnanthaceae | 8 | 12 |  |
| Setchellanthaceae | 1 | 12 |  |
| Caricaceae | 34 | 12 |  |
| Moringaceae | 12 | 12 |  |
| Tapisciaceae | 5 | 12 |  |
| Aphloiaceae | 1 | 12 |  |
| Ixerbaceae | 1 | 12 |  |
| Crossosomataceae | 12 | 12 |  |
| Stachyuraceae | 5 | 12 |  |
| Staphyleaceae | 45 | 12 |  |
| Melianthaceae | 13 | 12 | includes Francoaceae [= 2sp] |
| Geraniaceae | 805 | 12 |  |
| Vivianaceae | 12 | 12 | includes Ledocarpaceae [= 6 spp] |
| Cercidiphyllaceae | 2 | 12 |  |
| Crassulaceae | 1370 | 12 |  |
| Haloragaceae | 145 | 12 |  |
| Penthoraceae | 2 | 12 |  |
| Tetracarpaeaceae | 1 | 12 |  |
| Saxifragaceae | 540 | 12 |  |
| Iteaceae | 21 | 12 |  |
| Pterostemon | 3 | 12 |  |
| Grossulariaceae | 150 | 12 |  |
| Daphniphyllaceae | 10 | 12 |  |
| Paeoniaceae | 33 | 12 |  |
| Altingiaceae | 13 | 12 |  |
| Hamamelidaceae | 82 | 12 |  |
| Aextoxicaceae | 1 | 12 |  |
| Berberidopsidaceae | 3 | 12 |  |
| Loasaceae | 355 | 12 | includes sister taxon Hydrangeaceae [= 190sp] |
| Cornaceae | 107 | 12 |  |
| Grubbiaceae | 4 | 12 | includes sister taxon Curtisaceae [= 1sp] |
| Marcgraviaceae | 130 | 12 |  |
| Balsaminaceae | 1001 | 12 |  |
| Tetrameristaceae | 4 | 12 |  |
| Fouquieriaceae | 11 | 12 |  |
| Polemoniaceae | 385 | 12 |  |
| Lecythidaceae | 310 | 12 |  |
| Ebenaceae | 548 | 12 |  |
| Sapotaceae | 1100 | 12 |  |
| Theophrastaceae | 105 | 12 |  |
| Myrsinaceae | 1435 | 12 |  |
| Primulaceae | 900 | 12 |  |
| Pentaphylacaceae | 340 | 12 | includes Sladenaceae [= 3sp] |
| Actinidiaceae | 355 | 12 |  |
| Roridulaceae | 2 | 12 |  |
| Clethraceae | 75 | 12 |  |
| Cyrillaceae | 3997 | 12 | includes Ericaceae [= 3995sp] |
| Theaceae | 460 | 12 |  |
| Symplocaceae | 320 | 12 |  |
| Styracaceae | 178 | 12 | includes Diapensiaceae [= 18sp] |
| Icacinaceae_p.p. | 150 | 12 |  |
| Oncothecaceae | 2 | 12 |  |
| Garryaceae | 17 | 12 |  |
| Rubiaceae | 13150 | 12 |  |
| Apocynaceae | 4555 | 12 |  |
| Gentianaceae | 1655 | 12 |  |
| Gelsemiaceae | 11 | 12 |  |
| Loganiaceae | 420 | 12 |  |
| Boraginaceae | 2740 | 12 |  |
| Vahliaceae | 8 | 12 |  |
| Convolvulaceae | 1625 | 12 |  |
| Solanaceae | 2460 | 12 |  |
| Sphenocleaceae | 14 | 12 | includes Hydroleaceae [= 12sp] |
| Montiniaceae | 5 | 12 |  |
| Plocospermataceae | 1 | 12 |  |
| Oleaceae | 615 | 12 |  |
| Tetrachondraceae | 3 | 12 |  |
| Gesneriaceae | 3200 | 12 |  |
| Plantaginaceae | 1700 | 12 |  |
| Scrophulariaceae | 1700 | 12 |  |
| Phrymaceae | 234 | 12 |  |
| Paulowniaceae | 6 | 12 |  |
| Orobanchaceae | 2060 | 12 |  |
| Martyniaceae | 16 | 12 |  |
| Bignoniaceae | 800 | 12 |  |
| Lamiaceae | 7173 | 12 |  |
| Verbenaceae | 1175 | 12 |  |
| Pedaliaceae | 70 | 12 |  |
| Stilbaceae | 39 | 12 |  |
| Schlegeliaceae | 28 | 12 |  |
| Acanthaceae | 4000 | 12 |  |
| Cardiopteridaceae | 140 | 12 | includes Stemonuraceae [= 95sp] |
| Aquifoliaceae | 405 | 12 |  |
| Helwingiaceae | 3 | 12 |  |
| Phyllonomaceae | 4 | 12 |  |
| Torricelliaceae | 10 | 12 |  |
| Griseliniaceae | 6 | 12 |  |
| Araliaceae | 1450 | 12 |  |
| Apiaceae | 3780 | 12 |  |
| Pittosporaceae | 200 | 12 |  |
| Paracryphiaceae | 36 | 12 |  |
| Adoxaceae | 200 | 12 |  |
| Caprifoliaceae | 874 | 12 |  |
| Eremosynaceae | 1 | 13 |  |
| Escalloniaceae | 68 | 12 |  |
| Polyosmaceae | 60 | 14 |  |
| Tribelaceae | 1 | 13 |  |
| Bruniaceae | 75 | 12 |  |
| Columelliaceae | 5 | 12 |  |
| Pentaphragmataceae | 30 | 12 |  |
| Rousseaceae | 2393 | 12 | includes Campanulaceae [= 2380sp] |
| Stylidiaceae | 245 | 12 |  |
| Argophyllaceae | 21 | 12 |  |
| Alseuosmiaceae | 10 | 12 |  |
| Phellinaceae | 12 | 12 |  |
| Menyanthaceae | 58 | 12 |  |
| Goodeniaceae | 440 | 12 |  |
| Asteraceae | 23600 | 12 |  |
| Calyceraceae | 60 | 12 |  |
| Ancistrocladaceae | 12 | 12 |  |
| Dioncophyllaceae | 3 | 12 |  |
| Nepenthaceae | 90 | 12 |  |
| Polygonaceae | 1110 | 12 |  |
| Plumbaginaceae | 836 | 12 |  |
| Droseraceae | 115 | 12 |  |
| Frankeniaceae | 90 | 12 |  |
| Tamaricaceae | 90 | 12 |  |
| Asteropeiaceae | 10 | 12 | includes Physenaceae [= 2sp] |
| Nyctaginaceae | 397 | 12 | includes Sarcobataceae [= 2sp] |
| AizoaceaeXPhytolaccaceae | 2085 | 12 |  |
| Molluginaceae | 87 | 12 |  |
| Cactaceae | 1532 | 12 | includes Anacampserotacae [= 32sp] |
| Portulacaceae | 100 | 12 |  |
| Amaranthaceae | 2500 | 12 |  |
| Caryophyllaceae | 2200 | 12 |  |
| Simmondsiaceae | 1 | 12 |  |
| Rhabdodendraceae | 3 | 12 |  |
| Dilleniaceae | 300 | 12 |  |
| Loranthaceae | 1013 | 12 | includes Scoepfiaceae [= 55sp] and Misodendraceae [= 8sp] |
| Santalaceae | 990 | 12 |  |
| Opiliaceae | 28 | 13 |  |
| Olacaceae | 103 | 12 |  |
| Gunneraceae | 50 | 12 |  |
| Myrothamnaceae | 2 | 12 |  |
| Trochodendraceae | 2 | 12 |  |
| Buxaceae | 71 | 12 | includes Haptanthaceae [=1sp] |
| Didymelaceae | 2 | 12 |  |
| Sabiaceae | 100 | 12 |  |
| Nelumbonaceae | 2 | 12 |  |
| Platanaceae | 10 | 12 |  |
| Proteaceae | 1600 | 12 |  |
| Lardizabalaceae | 36 | 12 |  |
| Circaeasteraceae | 2 | 12 |  |
| Berberidaceae | 701 | 12 |  |
| Ranunculaceae | 2525 | 12 |  |
| Menispermaceae | 420 | 12 |  |
| Eupteleaceae | 2 | 12 |  |
| Papaveraceae | 760 | 12 |  |
| Poaceae | 10035 | 12 |  |
| Ecdeiocoleaceae | 3 | 12 |  |
| Joinvilleaceae | 2 | 12 |  |
| Restionaceae | 520 | 12 |  |
| Anarthriaceae | 11 | 12 |  |
| Centrolepidaceae | 35 | 12 |  |
| Flagellariaceae | 4 | 12 |  |
| Xyridaceae | 260 | 12 |  |
| Eriocaulaceae | 1160 | 12 |  |
| Bromeliaceae | 1700 | 12 |  |
| Juncaceae | 430 | 12 |  |
| Cyperaceae | 4350 | 12 |  |
| Thurniaceae | 4 | 12 |  |
| Typhaceae | 14 | 12 |  |
| Sparganiaceae | 13 | 12 |  |
| Rapateaceae | 94 | 12 |  |
| Zingiberaceae | 1300 | 12 |  |
| Costaceae | 110 | 12 |  |
| Marantaceae | 550 | 12 |  |
| Cannaceae | 10 | 12 |  |
| Streliziaceae | 7 | 12 |  |
| Lowiaceae | 15 | 12 |  |
| Musaceae | 35 | 12 |  |
| Heliconiaceae | 200 | 12 |  |
| Pontederiaceae | 33 | 12 |  |
| Commelinaceae | 652 | 12 |  |
| Haemodoraceae | 116 | 12 |  |
| Hanguanaceae | 6 | 12 |  |
| Philydraceae | 5 | 12 |  |
| Dasypogonaceae | 16 | 12 |  |
| Arecaceae | 2361 | 12 |  |
| Asparagaceae | 2648 | 12 | includes Laxmanniaceae [= 178sp], Ruscaceae [= 475sp], Agavaceae [= 637sp], Hyacinthaceae[= 1000sp], Themidaceae [=62sp], Aphyllanthaxeae [= 1sp] |
| Alliaceae | 1604 | 12 | includes Amaryllidaceae [= 800sp], Agapanthacaceae [= 9sp] |
| Xanthorrhoeaceae | 815 | 12 | includes Asphodelaceae [= 785sp] |
| Xeronemataceae | 2 | 12 |  |
| Iridaceae | 2025 | 12 |  |
| Doryanthaceae | 2 | 12 |  |
| Tecophilaeaceae | 23 | 12 |  |
| Ixiolirionaceae | 3 | 12 |  |
| Orchidaceae | 22075 | 12 |  |
| Hypoxidaceae | 220 | 12 |  |
| Blandfordaceae | 4 | 12 |  |
| Asteliaceae | 36 | 12 |  |
| Boryaceae | 12 | 12 |  |
| Lanariaceae | 1 | 12 |  |
| Colchicaceae | 245 | 12 |  |
| Alstroemeriaceae | 165 | 12 |  |
| Luzuriagaceae | 5 | 12 |  |
| Melanthiaceae | 170 | 12 |  |
| Rhipogonaceae | 6 | 12 |  |
| Philesiaceae | 2 | 12 |  |
| Smilaceae | 315 | 12 |  |
| Liliaceae | 610 | 12 |  |
| Campynemataceae | 4 | 12 |  |
| Velloziaceae | 240 | 12 |  |
| Stemonaceae | 75 | 12 | includes Triuridaceae [= 48sp] |
| Pandanaceae | 885 | 12 |  |
| Cyclanthaceae | 225 | 12 |  |
| Dioscoreaceae | 870 | 12 |  |
| Burmanniaceae | 95 | 12 |  |
| Nartheciaceae | 41 | 12 |  |
| Petrosaviaceae | 4 | 12 |  |
| Zosteraceae | 14 | 12 |  |
| Potamogetonaceae | 102 | 12 |  |
| Ruppiaceae | 10 | 12 |  |
| Posidoniaceae | 9 | 12 |  |
| Cymodoceaceae | 16 | 12 |  |
| Juncaginaceae | 15 | 12 |  |
| Scheuchzeriaceae | 1 | 12 |  |
| Aponogetonaceae | 43 | 12 |  |
| Hydrocharitaceae | 116 | 12 |  |
| Butomaceae | 1 | 12 |  |
| Limnocharitaceae | 12 | 13 |  |
| Alismataceae | 90 | 13 |  |
| Tofieldiaceae | 27 | 12 |  |
| Araceae | 4025 | 12 |  |
| Acoraceae | 4 | 12 |  |
| Sordariomycetes | 10564 | 15 |  |
| Eurotiomycetes | 3401 | 15 |  |
| Pezizomycetes | 1684 | 15 |  |
| Saccharomycetes | 915 | 15 |  |
| Taphrinomycetes | 140 | 15 |  |
| Schizosaccharomycetes | 5 | 15 |  |
| Agaricomycotina | 21429 | 15 |  |
| Ustilaginomycotina | 1113 | 15 | not resolved in time tree - treated as Basidiomycetes |
| Pucciniomycotina | 8324 | 15 |  |
| Glomeromycota | 169 | 15 |  |
| Mucoromycotina | 325 | 15 |  |
| Chytridiomycota | 706 | 15 |  |
| Blastocladiomycota | 179 | 15 |  |
| Neocallimastigomycota | 20 | 15 |  |
| Choanoflagellida | 125 | 16 |  |
| Hexactinnellida | 500 | 17 |  |
| Demospongiae | 6000 | 18 |  |
| Calcarea | 500 | 18 |  |
| Ctenophora | 190 | 19 |  |
| Cubozoa | 32 | 20 |  |
| Scyphozoa | 212 | 20 |  |
| Hydrozoa | 3500 | 21 |  |
| Platyhelmintha | 17000 | 22 |  |
| Nemertea | 1149 | 23 |  |
| Idiosepiidae | 8 | 24 |  |
| Bathyteuthidae | 3 | 24 |  |
| Pyroteuthidae | 6 | 24 |  |
| Joubiniteuthidae | 1 | 24 |  |
| Ommastrephidae | 21 | 24 |  |
| Loliginidae | 42 | 24 |  |
| Spirulidae | 1 | 24 |  |
| Sepiidae | 115 | 24 |  |
| Sepiolidae | 39 | 24 |  |
| Vampyroteuthidae | 1 | 24 |  |
| Stauroteuthidae | 2 | 24 |  |
| Opisthoteuthidae | 18 | 24 |  |
| Argonautidae | 7 | 24 |  |
| Tremoctopodidae | 3 | 24 |  |
| Octopodidae_1 | 125 | 24 |  |
| Octopodidae_2 | 32 | 24 |  |
| Octopodidae_3 | 11 | 24 |  |
| Vitreledonellidae | 1 | 24 |  |
| Bolitaenidae | 7 | 24 |  |
| Fustiariidae | 11 | 25 |  |
| Rhabdidae | 6 | 25 |  |
| Dentaliidae | 159 | 25 |  |
| Entalinidae | 35 | 25 |  |
| Gadilidae_1 | 53 | 25 |  |
| Pulsellidae | 18 | 25 |  |
| Gadilidae_2 | 130 | 25 |  |
| Annelida | 17000 | 26 |  |
| Rhabditidae | 23000 | 27 |  |
| Priapulida | 17 | 28 |  |
| Chilopoda | 3196 | 29 |  |
| Symphyla | 200 | 29 |  |
| Diplopoda | 11000 | 29 |  |
| Xiphosura | 4 | 30 |  |
| Ctenizidae | 122 | 31 |  |
| Cyrtaucheniidae | 134 | 31 |  |
| Idiopidae | 304 | 31 |  |
| Theraphosidae | 920 | 31 |  |
| Barychelidae | 300 | 31 |  |
| Microstigmatidae | 15 | 31 |  |
| Nemesiidae | 346 | 31 |  |
| Hexathelidae | 86 | 31 |  |
| Actinopodidae | 41 | 31 |  |
| Migidae | 91 | 31 |  |
| Dipluridae | 177 | 31 |  |
| Antrodiaetidae | 32 | 31 |  |
| Atypidae | 43 | 31 |  |
| Mecicobothriidae | 9 | 31 |  |
| Uloboridae | 266 | 31 |  |
| Deinopidae | 57 | 31 |  |
| Araneidae | 2990 | 31 |  |
| Theridiidae | 2295 | 31 |  |
| Zorocratidae | 42 | 31 |  |
| Pisauridae | 339 | 31 |  |
| Hypochilidae | 11 | 31 |  |
| Filistatidae | 111 | 31 |  |
| Diguetidae | 15 | 31 |  |
| Plectreuridae | 30 | 31 |  |
| Picnogonida | 1300 | 32 |  |
| Ostracoda | 8000 | 33 |  |
| Astacidea | 653 | 34 |  |
| Thalassinidea | 615 | 34 |  |
| Achelata | 140 | 34 |  |
| Anomala/Anomura | 2451 | 34 |  |
| Brachyura | 6559 | 34 |  |
| Caridea | 3268 | 34 |  |
| Stenopodidae | 69 | 34 |  |
| Penaeidae | 540 | 34 |  |
| Poecilasmatidae | 22 | 35 |  |
| Heteralepadidae_1 | 6 | 35 |  |
| Oxynaspididae | 3 | 35 |  |
| Lepadidae | 12 | 35 |  |
| Heteralepadidae_2 | 2 | 35 |  |
| Neoverrucidae | 2 | 36 |  |
| Eolepadidae | 4 | 35 |  |
| Scalpellidae | 125 | 35 |  |
| Tetraclitoidea | 19 | 35 |  |
| Balanoidea_1 | 63 | 35 | all other Balanoidea |
| Coronuloidea | 14 | 35 |  |
| Balanoidea_2 | 3 | #VALUE! | Elminius |
| Chthamaloidea | 12 | 35 |  |
| Verrucidae | 21 | 35 |  |
| Lithotryidae | 1 | 35 |  |
| Pollicipedidae | 1 | 35 |  |
| Calanticidae | 3 | 35 |  |
| Iblidae | 9 | 35 |  |
| Cephalocarida | 10 | 38 |  |
| Remipedia | 20 | 39 |  |
| Branchiopoda | 794 | 33 |  |
| Mecoptera | 500 | 33 |  |
| Siphonaptera | 1750 | 33 |  |
| Mycetophilidae_1 | 3817 | 35 |  |
| Sciaridae | 2103 | 35 |  |
| Mycetophilidae_2 | 876 | 35 |  |
| Cecidomyiidae | 6064 | 35 |  |
| Bibionidae | 804 | 35 |  |
| Pachyneuridae | 8 | 35 |  |
| Mycetophilidae_3 | 64 | 35 |  |
| Scatopsidae | 301 | 35 |  |
| Canthyloscelidae | 17 | 35 |  |
| Anisopodidae | 184 | 35 |  |
| Xylophagidae | 83 | 35 |  |
| Vermileonidae | 57 | 35 |  |
| Rhagionidae | 440 | 35 |  |
| Pelecorhynchidae | 4 | 35 |  |
| Tabanidae | 4283 | 35 |  |
| Stratiomyidae | 2704 | 35 |  |
| Xylomyidae | 130 | 35 |  |
| Pantophthalmidae | 21 | 35 |  |
| Nemestrinidae | 268 | 35 |  |
| Acroceridae | 398 | 35 |  |
| Bombyliidae | 5009 | 35 |  |
| Apioceridae | 138 | 35 |  |
| Mydidae | 461 | 35 |  |
| Asilidae | 6919 | 35 |  |
| Scenopinidae | 388 | 35 |  |
| Therevidae | 1032 | 35 |  |
| Atelestidae | 9 | 35 |  |
| Empididae | 4752 | 35 |  |
| Dolichopodidae | 6661 | 35 |  |
| Platypezidae | 265 | 35 |  |
| Syrphidae | 5785 | 35 |  |
| Drosophilidae | 3833 | 35 |  |
| Muscidae | 5267 | 35 |  |
| Perissommatidae | 7 | 35 |  |
| Psychodidae | 398 | 35 |  |
| Tanyderidae | 45 | 35 |  |
| Blephariceridae | 331 | 35 |  |
| Culicidae | 3532 | 35 |  |
| Chaoboridae | 78 | 35 |  |
| Corethrellidae | 67 | 35 |  |
| Dixidae | 175 | 35 |  |
| Ceratopogonidae | 5654 | 35 |  |
| Chironomidae | 7812 | 35 |  |
| Simuliidae | 1923 | 35 |  |
| Thaumaleidae | 170 | 35 |  |
| Nymphomyiidae | 8 | 35 |  |
| Axymylidae | 6 | 35 |  |
| Ptychopteridae | 75 | 35 |  |
| Tipulidae | 4297 | 35 |  |
| Trichoceridae | 162 | 35 |  |
| Deuterophelbiidae | 14 | 35 |  |
| Strepsiptera | 532 | 40 |  |
| Lepidoptera | 174250 | 41 |  |
| Trichoptera | 13574 | 42 |  |
| Megaloptera | 300 | 43 |  |
| Rhaphidioptera | 206 | 44 |  |
| Hemerobiidae | 1140 | 45 |  |
| Chrysopidae | 3059 | 45 |  |
| Ithonidae | 22 | 45 |  |
| Polystoechotidae | 4 | 45 |  |
| Nemopteridae | 265 | 45 |  |
| Myrmeleontidae | 3353 | 45 |  |
| Ascalaphidae | 751 | 45 |  |
| Nymphidae | 42 | 45 |  |
| Psychopsidae | 47 | 45 |  |
| Mantispidae | 714 | 45 |  |
| Berothidae | 9 | 45 |  |
| Rhachiberothidae | 12 | 45 |  |
| Osmylidae | 314 | 45 |  |
| Nevrorthidae | 16 | 45 |  |
| Sisyridae | 72 | 45 |  |
| Dilaridae | 101 | 45 |  |
| Coniopterygidae | 896 | 45 |  |
| Pyrochroidae_1 | 200 | 0 |  |
| Pythidae | 20 | 0 |  |
| Salpingidae_1 | 150 | 0 |  |
| Melandryidae_1 | 380 | 0 |  |
| Tetratomidae_1 | 11 | 0 |  |
| Trictenotomidae | 15 | 0 |  |
| Scraptiidae_1 | 300 | 0 |  |
| Melandryidae_2 | 50 | 0 |  |
| Scraptiidae_2 | 100 | 0 |  |
| Boridae | 4 | 0 |  |
| Oedemeridae | 1500 | 0 |  |
| Tetratomidae_2 | 22 | 0 |  |
| Perimylopidae | 19 | 0 |  |
| Melandryidae_3 | 55 | 0 |  |
| Zopheridae_1 | 127 | 0 |  |
| Pyrochroidea_2 | 1 | 0 |  |
| Monommatidae | 300 | 0 |  |
| Salpingidae_2 | 20 | 0 |  |
| Mycetophagidae | 200 | 0 |  |
| Tenebrionidae_1 | 10000 | 0 |  |
| Melandryidae_4 | 100 | 0 |  |
| Stenotrachelidae | 20 | 0 | Cephaloinae |
| Zopheridae_2 | 1000 | 0 |  |
| Salpingidae_3 | 50 | 0 |  |
| Anthicidae_1 | 50 | 0 |  |
| Aderidae | 1000 | 0 |  |
| Tenebrionidae_2 | 1600 | 0 |  |
| Tenebrionidae_3 | 14000 | 0 |  |
| Meloidae | 1805 | 0 |  |
| Anthicidae_2 | 2900 | 0 |  |
| Anthicidae_3 | 125 | 0 |  |
| Ciidae | 550 | 0 |  |
| Rhipiphoridae | 395 | 0 |  |
| Lymexylidae_1 | 30 | 0 |  |
| Mordellidae | 1500 | 0 |  |
| Lymexylidae_2 | 24 | 0 |  |
| Sphindidae | 60 | 0 |  |
| Corylophidae | 280 | 0 |  |
| Endomychidae_1 | 50 | 0 |  |
| Endomychidae_2 | 100 | 0 |  |
| Endomychidae_3 | 750 | 0 |  |
| Latridiidae | 1050 | 0 |  |
| Coccinellidae | 6081 | 0 |  |
| Endomychidae_4 | 500 | 0 |  |
| Alexiidae | 32 | 0 |  |
| Cerylonidae_1 | 60 | 0 |  |
| Bothrideridae_1 | 30 | 0 |  |
| Bothrideridae_2 | 50 | 0 |  |
| Discolomatidae | 400 | 0 |  |
| Bothrideridae_3 | 3 | 0 |  |
| Cerylonidae_2 | 740 | 0 |  |
| Melyridae_1 | 5050 | 0 |  |
| Prionoceridae | 150 | 0 |  |
| Melyridae_2 | 300 | 0 |  |
| Trogossitidae_1 | 104 | 0 |  |
| Cleridae | 3600 | 0 |  |
| Trogossitidae_2 | 20 | 0 |  |
| Byturidae | 16 | 0 |  |
| Biphyllidae | 195 | 0 |  |
| Phloiophilidae | 1 | 0 |  |
| Trogossitidae_3 | 392 | 0 |  |
| Erotylidae | 2500 | 0 |  |
| Helotidae | 100 | 0 |  |
| Protocucujidae | 5 | 0 |  |
| Monotomidae | 200 | 0 |  |
| Nitidulidae | 2274 | 0 |  |
| Laemophloeidae | 400 | 0 |  |
| Propalticidae | 35 | 0 |  |
| Phalacridae | 504 | 0 |  |
| Cryptophagidae | 600 | 0 |  |
| Passandridae | 105 | 0 |  |
| Cucujidae | 20 | 0 |  |
| Megalopodidae_1 | 55 | 0 |  |
| Cerambycidae_1 | 30 | 0 |  |
| Cerambycidae_2 | 100 | 0 |  |
| Orsodacnidae_1 | 10 | 0 |  |
| Vesperidae | 50 | 0 |  |
| Megalopodidae_2 | 4 | 0 |  |
| Orsodacnidae_2 | 19 | 0 |  |
| Cerambycidae_3 | 2575 | 0 |  |
| Disteniidae | 80 | 0 |  |
| Chrysomelidae_1 | 25919 | 0 |  |
| Cerambycidae_4 | 18600 | 0 |  |
| Curculionidae_1 | 44236 | 0 |  |
| Ithyceridae | 1 | 0 |  |
| Brentidae_1 | 266 | 0 |  |
| Curculionidae_2 | 385 | 0 |  |
| Brentidae_2 | 2100 | 0 |  |
| Caridae | 5 | 0 |  |
| Brentidae_3 | 38 | 0 |  |
| Curculionidae_3 | 5800 | 0 |  |
| Brentidae_4 | 1165 | 0 |  |
| Belidae | 175 | 0 |  |
| Anthribidae_1 | 3000 | 0 |  |
| Anthribidae_2 | 80 | 0 |  |
| Nemonychidae_1 | 20 | 0 |  |
| Attelabidae | 2052 | 0 |  |
| Nemonychidae_2 | 15 | 0 |  |
| Chrysomelidae_2 | 6000 | 0 |  |
| Silvanidae | 470 | 0 |  |
| Phloeostichidae | 14 | 40 |  |
| Lycidae | 4500 | 0 |  |
| Lampyridae | 800 | 0 | Luciolinae plus Ototretinae |
| Cantharidae | 3300 | 0 |  |
| Elateridae_1 | 3720 | 0 |  |
| Elateridae_2 | 800 | 0 |  |
| Phengodidae | 280 | 0 |  |
| Rhagophthalmidae | 150 | 0 |  |
| Elateridae_3 | 750 | 0 |  |
| Drilidae | 220 | 0 |  |
| Elateridae_4 | 2300 | 0 |  |
| Omalisidae | 10 | 0 |  |
| Eucnemidae | 1300 | 0 |  |
| Throscidae | 152 | 0 |  |
| Telegeusidae | 8 | 0 |  |
| Omethidae | 22 | 40 |  |
| Dascillidae | 80 | 0 |  |
| Rhipiceridae | 57 | 0 |  |
| Byrrhidae | 400 | 0 |  |
| Nosodendridae | 70 | 0 |  |
| Ptilodactylidae | 510 | 0 |  |
| Callirhipidae | 16 | 0 |  |
| Chelonariidae | 300 | 0 |  |
| Elmidae_1 | 1200 | 0 |  |
| Elmidae_2 | 130 | 0 |  |
| Eulichadidae | 23 | 0 |  |
| Psephenidae | 58 | 0 | Eubrianacinae |
| Limnichidae | 376 | 0 |  |
| Dryopidae | 280 | 0 |  |
| Buprestidae | 14000 | 0 |  |
| Heteroceridae | 349 | 0 |  |
| Anobiidae_1 | 2520 | 0 |  |
| Bostrichidae_1 | 450 | 0 |  |
| Anobiidae_2 | 84 | 0 |  |
| Bostrichidae_2 | 90 | 0 |  |
| Dermestidae | 1230 | 0 |  |
| Scarabaeidae_1 | 20450 | 0 |  |
| Glaphyridae | 174 | 0 |  |
| Ochodaeidae | 105 | 0 |  |
| Scarabaeidae_2 | 5317 | 0 |  |
| Glaresidae | 50 | 0 |  |
| Hybosoridae | 217 | 0 |  |
| Scarabaeidae_3 | 3187 | 0 |  |
| Lucanidae | 1171 | 0 |  |
| Ceratocanthidae | 307 | 0 |  |
| Bolboceratidae | 300 | 0 |  |
| Passalidae | 675 | 0 |  |
| Trogidae | 330 | 0 |  |
| Staphylinidae_1 | 19804 | 0 |  |
| Staphylinidae_2 | 1519 | 0 |  |
| Silphidae | 113 | 0 |  |
| Staphylinidae_3 | 15352 | 0 |  |
| Hydrophilidae | 2804 | 0 |  |
| Leiodidae | 3290 | 0 |  |
| Agyrtidae | 61 | 0 |  |
| Hydraenidae | 1094 | 0 |  |
| Ptilidae | 628 | 0 |  |
| Histeridae | 3841 | 0 |  |
| Sphaeritidae | 4 | 0 |  |
| Synteliidae | 9 | 0 |  |
| Scydmaenidae | 4500 | 0 |  |
| Clambidae | 150 | 0 |  |
| Eucinetidae | 37 | 0 |  |
| Derodontidae | 17 | 0 |  |
| Decliniidae | 2 | 0 |  |
| Scirtidae | 1000 | 0 |  |
| Amphizoidae | 5 | 0 |  |
| Dytiscidae_1 | 1956 | 0 |  |
| Dytiscidae_2 | 1826 | 0 |  |
| Hygrobiidae | 6 | 40 |  |
| Aspidytidae | 2 | 0 |  |
| Noteridae | 235 | 0 |  |
| Haliplidae | 204 | 0 |  |
| Gyrinidae | 1100 | 0 |  |
| Rhysodidae_AND_Carabida_1 | 30262 | 0 |  |
| Brachinidae | 655 | 0 |  |
| Carabidae_2 | 84 | 0 |  |
| Trachypachidae | 4 | 0 |  |
| Carabidae_3 | 49 | 0 |  |
| Torridincolidae | 60 | 0 |  |
| Hydroscaphidae | 13 | 0 |  |
| Cupedidae | 30 | 0 |  |
| Sphaeriusidae | 23 | 0 |  |
| Colletidae | 2250 | 46 |  |
| Stenotritidae | 21 | 46 |  |
| Halictidae | 2287 | 46 |  |
| Andrenidae | 2207 | 46 |  |
| Apidae | 4919 | 46 |  |
| Megachilidae | 963 | 46 |  |
| Melittidae_s.s. | 30 | 46 |  |
| Dasypodaidae | 38 | 46 |  |
| Crabronidae | 1232 | 46 |  |
| Sphecidae | 6432 | 46 |  |
| Sapygidae | 17 | 46 |  |
| Mutillidae | 1238 | 46 |  |
| Pompilidae | 299 | 46 |  |
| Tiphiidae | 92 | 46 |  |
| Vespidae | 4447 | 46 |  |
| Scoliidae | 32 | 46 |  |
| Formicidae | 12571 | 46 |  |
| Bradynobaenidae | 70 | 46 |  |
| Rhopalosomatidae | 3 | 46 |  |
| Bethylidae | 1847 | 46 |  |
| Chrysididae | 2395 | 46 |  |
| Dryinidae | 845 | 46 |  |
| Paraneoptera | 1489 | 47 |  |
| Hemichordata | 110 | 48 |  |
| Echinodermata | 7000 | 33 |  |
| Urochordata | 85 | 49 |  |
| Cephalochordata | 20 | 49 |  |
| Myxinidae | 67 | 49 |  |
| Petromyzonidae | 41 | 49 |  |
| Carcharhinidae | 50 | 49 |  |
| Hemigaleidae | 7 | 49 |  |
| Triakidae_1 | 27 | 49 |  |
| Triakidae_2 | 5 | 49 |  |
| Leptochariidae | 1 | 49 |  |
| Pseudotriakidae | 1 | 49 |  |
| Proscylliidae | 6 | 49 |  |
| Scyliorhinidae_1 | 54 | 49 |  |
| Scyliorhinidae_2 | 35 | 49 |  |
| Alopiidae | 3 | 49 |  |
| Megachasmidae | 1 | 49 |  |
| Odontaspididae | 4 | 49 |  |
| Pseudocarchariidae | 1 | 49 |  |
| Lamnidae | 5 | 49 |  |
| Cetorhinidae | 1 | 49 |  |
| Carchariidae | 1 | 49 |  |
| Mitsukurinidae | 1 | 49 |  |
| Ginglymostomidae | 3 | 49 |  |
| Stegosomatidae | 1 | 49 |  |
| Hemiscylliidae | 11 | 49 |  |
| Brachaeluridae | 2 | 49 |  |
| Orectolobidae | 6 | 49 |  |
| Parascylliidae | 7 | 49 |  |
| Heterodontidae | 8 | 49 |  |
| Somniosidae | 17 | 49 |  |
| Oxynotidae | 5 | 49 |  |
| Dalatiidae | 49 | 49 |  |
| Etmopteridae | 41 | 49 |  |
| Centrophoridae | 13 | 49 |  |
| Squalidae | 10 | 49 |  |
| Squatinidae | 13 | 49 |  |
| Echinorhinidae | 2 | 49 |  |
| Pristiophoridae | 5 | 49 |  |
| Notorynchidae | 3 | 49 | Hexachidae |
| Hexanchidae | 4 | 49 |  |
| Chlaydoselachidae | 1 | 49 |  |
| Myliobatidae | 42 | 49 |  |
| Gymnuridae | 12 | 49 |  |
| Dasyatidae_1 | 40 | 49 |  |
| Dasyatidae_2 | 31 | 49 |  |
| Urolophidae | 35 | 49 |  |
| Hexatrygonidae | 1 | 49 |  |
| Plesiobatidae | 1 | 49 |  |
| Potamotrygonidae | 20 | 49 |  |
| Urotrygonidae | 16 | 49 |  |
| Rhinidae | 6 | 49 |  |
| Rhynchobatidae | 39 | 49 |  |
| Pristidae | 4 | 49 |  |
| Rhinobatidae | 45 | 49 |  |
| Platyrhinidae | 4 | 49 |  |
| Torpedinidae | 21 | 49 |  |
| Narcinidae_1 | 30 | 49 |  |
| Narcinidae_2 | 10 | 49 |  |
| Rajidae | 200 | 49 |  |
| Rhinochimaeridae | 6 | 49 |  |
| Chimaeridae | 20 | 49 |  |
| Callorhinchidae | 4 | 49 |  |
| Lepisosteiformes | 7 | 49 |  |
| Acipenseridae | 23 | 49 |  |
| Polyodontidae | 2 | 49 |  |
| Amiiformes | 1 | 49 |  |
| Siluriformes | 6791 | 49 |  |
| Clupeiformes | 397 | 49 |  |
| Gonorhynchiformes | 27 | 49 |  |
| Perciformes | 10391 | 49 |  |
| Salmoniformes | 66 | 49 |  |
| Anguilliformes | 893 | 49 |  |
| Osteoglossidae | 5 | 49 |  |
| Polypteriformes | 10 | 49 |  |
| Actinistia | 2 | 49 |  |
| Lepidosirenidae | 1 | 50 |  |
| Protopteridae | 4 | 50 |  |
| Ceratodontidae | 1 | 50 |  |
| Rhinatrematidae | 9 | 50 |  |
| Uraeotyphlidae | 9 | 50 |  |
| Ichthyophiidae | 44 | 50 |  |
| Scolecomorphidae | 6 | 50 |  |
| Caeciliidae_1 | 115 | 50 |  |
| Microhylidae | 85 | 50 |  |
| Dyscophidae | 3 | 50 |  |
| Asterophryidae | 236 | 50 |  |
| Kalophrynidae | 15 | 50 |  |
| Cophylidae | 46 | 50 |  |
| Melanobatrachidae | 1 | 50 |  |
| Gastrophrynidae | 43 | 50 |  |
| Scaphiophrynidae | 12 | 50 |  |
| Hoplophrynidae | 4 | 50 |  |
| Phrynomeridae | 5 | 50 |  |
| Arthroleptidae | 140 | 50 |  |
| Astylosternidae | 26 | 50 |  |
| Hyperoliidae | 210 | 50 |  |
| Brevicipitidae | 27 | 50 |  |
| Hemisotidae | 9 | 50 |  |
| Mantellidae | 174 | 50 |  |
| Rhacophoridae | 317 | 50 |  |
| Dicroglossidae | 150 | 50 |  |
| Ranidae | 539 | 50 |  |
| Nyctibatrachidae | 17 | 50 |  |
| Micrixalidae | 11 | 50 |  |
| Ranixalidae | 10 | 50 |  |
| Ceratobatrachidae | 83 | 50 |  |
| Petropedetidae | 16 | 50 |  |
| Pyxicephalidae | 54 | 50 |  |
| Phrynobatrachidae | 78 | 50 |  |
| Ptycephalidae | 51 | 50 |  |
| Sooglossidae | 4 | 50 |  |
| Nasikabatrachidae | 1 | 50 |  |
| Pelodryadidae | 151 | 50 |  |
| Phyllomedusidae | 61 | 50 |  |
| Brachycephalidae | 42 | 50 |  |
| Craugastoridae | 114 | 50 |  |
| Eleutherodactylidae | 202 | 50 |  |
| Strabomantidae | 555 | 50 |  |
| Hylidae | 663 | 50 |  |
| Ceratophryidae | 20 | 50 |  |
| Telmatobiidae | 68 | 50 |  |
| Bufonidae | 541 | 50 |  |
| Dendrobatidae | 178 | 50 |  |
| Leptodactylidae | 99 | 50 |  |
| Centrolenidae | 152 | 50 |  |
| Rhinodermatidae | 2 | 50 |  |
| Limnodynastidae | 44 | 50 |  |
| Rheobatrachidae | 2 | 50 |  |
| Myobatrachidae | 128 | 50 |  |
| Calyptocephalellidae | 4 | 50 |  |
| Heleophrynidae | 6 | 50 |  |
| Pelobatidae | 4 | 50 |  |
| Megophryidae | 140 | 50 |  |
| Pelodytidae | 3 | 50 |  |
| Scaphiopodidae | 7 | 50 |  |
| Pipidae | 30 | 50 |  |
| Rhinophrynidae | 1 | 50 |  |
| Alytidae | 5 | 50 |  |
| Discoglossidae | 7 | 50 |  |
| Bombinatoridae | 10 | 50 |  |
| Ascaphidae | 2 | 50 |  |
| Leiopelmatidae | 4 | 50 |  |
| Plethodontidae | 394 | 50 |  |
| Amphiumidae | 3 | 50 |  |
| Rhyacotritonidae | 4 | 50 |  |
| Proteidae | 6 | 50 |  |
| Ambystomatidae | 32 | 50 |  |
| Dicamptodontidae | 4 | 50 |  |
| Salamandridae | 81 | 50 |  |
| Sirenidae | 4 | 50 |  |
| Hynobiidae | 52 | 50 |  |
| Cryptobranchidae | 3 | 50 |  |
| Dibamidae | 21 | 51 |  |
| Sphaerodactylidae | 98 | 51 |  |
| Gekkonidae | 950 | 51 |  |
| Phyllodactylidae | 49 | 51 |  |
| Eublepharidae | 28 | 51 |  |
| Diplodactylidae | 125 | 51 |  |
| Carphodactylidae | 1 | 51 |  |
| Pygopodidae | 39 | 51 |  |
| Scincidae | 1425 | 51 |  |
| Xantusiidae | 29 | 51 |  |
| Gerrhosauridae | 34 | 51 |  |
| Cordylidae | 55 | 51 |  |
| Amphisbaenidae | 153 | 51 |  |
| Trogonophidae | 6 | 51 |  |
| Bipedidae | 3 | 51 |  |
| Blanidae | 4 | 51 |  |
| Cadeidae | 2 | 51 |  |
| Rhineuridae | 1 | 51 |  |
| Lacertidae | 301 | 51 |  |
| Teiidae | 123 | 51 |  |
| Gymnophthalmidae | 216 | 51 |  |
| Iguanidae | 37 | 51 |  |
| Chamaeleonidae | 178 | 51 |  |
| Agamidae | 416 | 51 |  |
| Anguidae | 64 | 51 |  |
| Anniellidae | 2 | 51 |  |
| Diploglossidae | 50 | 51 |  |
| Helodermatidae | 2 | 51 |  |
| Xenosauridae | 6 | 51 |  |
| Lanthanotidae | 1 | 51 |  |
| Varanidae | 68 | 51 |  |
| Shinisauridae | 1 | 51 |  |
| Dipsadidae | 416 | 51 |  |
| Pseudoxenodontidae | 10 | 51 |  |
| Colubridae | 628 | 51 |  |
| Natricidae | 204 | 51 |  |
| Elapidae | 153 | 51 |  |
| Lamprophiidae | 64 | 51 |  |
| Homalopsidae | 36 | 51 |  |
| Viperidae | 285 | 51 |  |
| Pareatidae | 15 | 51 |  |
| Xenodermatidae | 18 | 51 |  |
| Acrochordidae | 3 | 51 |  |
| Pythonidae | 35 | 51 |  |
| Loxocemidae | 1 | 51 |  |
| Xenopeltidae | 2 | 51 |  |
| Boidae | 78 | 51 |  |
| Uropeltidae | 47 | 51 |  |
| Bolyeriidae | 2 | 51 |  |
| Tropidophiidae | 28 | 51 |  |
| Aniliidae | 1 | 51 |  |
| Anomalepididae | 17 | 51 |  |
| Typhlopidae | 362 | 51 |  |
| Leptotyphlopidae | 107 | 51 |  |
| Sphenodontia | 2 | 51 |  |
| Geoemydidae | 70 | 51 |  |
| Testudinidae | 48 | 51 |  |
| Emydidae | 113 | 51 |  |
| Platysternidae | 1 | 51 |  |
| Kinosternidae | 25 | 51 |  |
| Dermatemydidae | 1 | 51 |  |
| Chelydridae | 3 | 51 |  |
| Dermochelyidae | 1 | 51 |  |
| Cheloniidae | 6 | 51 |  |
| Trionychidae | 30 | 51 |  |
| Carettochelyidae | 1 | 51 |  |
| Chelidae | 59 | 51 |  |
| Pelomedusidae | 19 | 51 |  |
| Podocnemidae | 8 | 51 |  |
| Gavialidae | 2 | 51 |  |
| Crocodylidae | 13 | 51 |  |
| Alligatoridae | 8 | 51 |  |
| Apterygidae | 5 | 52 |  |
| Casuariidae | 4 | 52 |  |
| Struthionidae | 1 | 52 |  |
| Rheidae | 2 | 52 |  |
| Tinamidae | 47 | 52 |  |
| Odontophoridae | 32 | 53 |  |
| Phasianidae | 178 | 53 |  |
| Numididae | 6 | 53 |  |
| Cracidae | 50 | 53 |  |
| Megapodiidae | 21 | 53 |  |
| Anatidae | 150 | 53 |  |
| Dendrocygnidae | 8 | 53 |  |
| Anseranatidae | 1 | 53 |  |
| Anhimidae | 3 | 53 |  |
| Phoenicopterformes | 6 | 54 |  |
| Podicipediformes | 20 | 54 |  |
| Gaviiformes | 5 | 54 |  |
| Procellariiformes | 125 | 55 |  |
| Sphenisciformes | 18 | 54 |  |
| Hemiprocnidae | 4 | 56 |  |
| Apodidae | 341 | 56 |  |
| Trochilidae | 347 | 56 |  |
| Trochiliformes | 331 | 54 |  |
| Caprimulgiformes | 113 | 54 |  |
| Pelecaniformes | 67 | 54 |  |
| Ciconiformes | 127 | 54 |  |
| Laridae | 95 | 54 |  |
| Rhynchopidae | 3 | 54 |  |
| Sternidae | 44 | 54 |  |
| Alcidae | 23 | 54 |  |
| Stercorariidae | 8 | 54 |  |
| Glareolidae | 17 | 54 |  |
| Turnicidae | 16 | 54 |  |
| Jacanidae | 8 | 54 |  |
| Rostratulidae | 2 | 54 |  |
| Thinocoridae | 4 | 54 |  |
| Pedionomidae | 1 | 54 |  |
| Scolopacidae | 89 | 54 |  |
| Haematopodidae | 11 | 54 |  |
| Ibidorhynchidae | 1 | 54 |  |
| Recurvirostridae | 9 | 54 |  |
| Pluvialidae | 4 | 54 |  |
| Charadriidae | 66 | 54 |  |
| Pluvianidae | 1 | 54 |  |
| Pluvianellidae | 1 | 54 |  |
| Chionidae | 2 | 54 |  |
| Burhinidae | 9 | 54 |  |
| Eurypygidae | 1 | 54 |  |
| Rhynochetidae | 1 | 54 |  |
| Heliornithidae | 3 | 54 |  |
| Rallidae | 136 | 54 |  |
| Psophiidae | 3 | 54 |  |
| Aramidae | 1 | 54 |  |
| Gruidae | 15 | 54 |  |
| Accipitridae | 240 | 57 |  |
| Pandionidae | 1 | 57 |  |
| Sagittariidae | 1 | 57 |  |
| Falconidae | 64 | 57 |  |
| Cathartidae | 7 | 57 |  |
| Ramphastidae | 36 | 58 |  |
| Capitonidae | 13 | 58 |  |
| Lybiidae | 42 | 58 |  |
| Megalaimidae | 26 | 58 |  |
| Indicatoridae | 17 | 58 |  |
| Picidae | 216 | 58 |  |
| Bucconidae | 35 | 58 |  |
| Galbulidae | 18 | 58 |  |
| Coraciiformes | 218 | 54 |  |
| Aegithalidae | 10 | 54 |  |
| Hirundinidae | 83 | 54 |  |
| Cisticolidae | 114 | 54 |  |
| Timaliidae | 324 | 54 |  |
| Zosteropidae | 98 | 54 |  |
| Sylviidae | 290 | 54 |  |
| Pycnonotidae | 124 | 54 |  |
| Alaudidae | 93 | 54 |  |
| Stenostiridae | 8 | 54 |  |
| Paridae | 53 | 54 |  |
| Promeropidae | 2 | 54 |  |
| Cardinalidae | 35 | 54 |  |
| Thraupidae | 271 | 54 |  |
| Emberizidae | 317 | 54 |  |
| Icteridae | 99 | 54 |  |
| Parulidae | 117 | 54 |  |
| Fringillidae | 165 | 54 |  |
| Motacillidae | 61 | 54 |  |
| Passeridae | 44 | 54 |  |
| Ploceidae | 106 | 54 |  |
| Prunellidae | 13 | 54 |  |
| Irenidae | 2 | 54 |  |
| Dicaeidae | 45 | 54 |  |
| Nectariniidae | 123 | 54 |  |
| Bombycillidae | 4 | 54 |  |
| Ptilogonatidae | 4 | 54 |  |
| Turdidae | 169 | 54 |  |
| Muscicapidae | 277 | 54 |  |
| Cinclidae | 5 | 54 |  |
| Mimidae | 35 | 54 |  |
| Sturnidae | 109 | 54 |  |
| Regulidae | 6 | 54 |  |
| Certhiidae | 10 | 54 |  |
| Sittidae | 25 | 54 |  |
| Polioptilidae | 15 | 54 |  |
| Troglodytidae | 79 | 54 |  |
| Petroicidae | 44 | 54 |  |
| Picathartidae | 2 | 54 |  |
| Aegithinidae | 4 | 54 |  |
| Malaconotidae | 45 | 54 |  |
| Prionopidae | 11 | 54 |  |
| Vangidae | 22 | 54 |  |
| Platysteiridae | 31 | 54 |  |
| Artamidae | 11 | 54 |  |
| Cracticidae | 12 | 54 |  |
| Rhipiduridae | 42 | 54 |  |
| Corcoracidae | 2 | 54 |  |
| Melampittidae | 2 | 54 |  |
| Paradisaeidae | 40 | 54 |  |
| Grallinidae | 2 | 54 |  |
| Monarchidae | 89 | 54 |  |
| Laniidae | 30 | 54 |  |
| Corvidae | 116 | 54 |  |
| Dicruridae | 23 | 54 |  |
| Oriolidae | 30 | 54 |  |
| Paramythiidae | 2 | 54 |  |
| Colluricinclidae | 14 | 54 |  |
| Pachycephalidae | 37 | 54 |  |
| Falcunculidae | 2 | 54 |  |
| Campephagidae | 82 | 54 |  |
| Vireonidae | 52 | 54 |  |
| Daphoenosittidae | 2 | 54 |  |
| Cnemophilidae | 3 | 54 |  |
| Callaeatidae | 2 | 54 |  |
| Melanocharitidae | 12 | 54 |  |
| Orthonychidae | 4 | 54 |  |
| Pomatostomidae | 5 | 54 |  |
| Meliphagidae | 176 | 54 |  |
| Pardalotidae | 4 | 54 |  |
| Maluridae | 27 | 54 |  |
| Ptilonorhynchidae | 19 | 54 |  |
| Climacteridae | 7 | 54 |  |
| Menuridae | 2 | 54 |  |
| Dendrocolaptidae | 52 | 54 |  |
| Furnariidae | 238 | 54 |  |
| Formicariidae | 63 | 54 |  |
| Rhinocryptidae | 57 | 54 |  |
| Conopophagidae | 8 | 54 |  |
| Thamnophilidae | 215 | 54 |  |
| Tyrannidae | 412 | 54 |  |
| Tityridae | 31 | 54 |  |
| Cotingidae | 96 | 54 |  |
| Pipridae | 54 | 54 |  |
| Philepittidae | 4 | 54 |  |
| Eurylaimidae | 15 | 54 |  |
| Pittidae | 31 | 54 |  |
| Acanthisittidae | 2 | 54 |  |
| Psittaciformes | 371 | 54 |  |
| Coliiformes | 6 | 54 |  |
| Trogoniformes | 39 | 54 |  |
| Tytonidae | 15 | 59 |  |
| Strigidae | 187 | 59 |  |
| Musophagiformes | 23 | 54 |  |
| Cuculiformes | 143 | 54 |  |
| Columbiformes | 313 | 54 |  |
| Tachyglossidae | 4 | 60 |  |
| Ornithorhynchidae | 1 | 60 |  |
| Potoroidae | 8 | 60 |  |
| Macropodidae | 65 | 60 |  |
| Hypsiprymnodontidae | 1 | 60 |  |
| Burramyidae | 5 | 60 |  |
| Phalangeridae | 27 | 60 |  |
| Acrobatidae | 2 | 60 |  |
| Petauridae | 11 | 60 |  |
| Pseudocheiridae | 17 | 60 |  |
| Tarsipedidae | 1 | 60 |  |
| Phascolarctidae | 1 | 60 |  |
| Vombatidae | 3 | 60 |  |
| Dasyuridae | 69 | 60 |  |
| Myrmecobiidae | 1 | 60 |  |
| Peramelidae | 18 | 60 |  |
| Thylacomyidae | 2 | 60 |  |
| Notoryctidae | 2 | 60 |  |
| Microbiotheridae | 1 | 60 |  |
| Caenolestidae | 6 | 60 |  |
| Caluromyidae | 4 | 60 |  |
| Didelphidae | 96 | 60 |  |
| Sirenia | 5 | 60 |  |
| Hyracoidea | 4 | 60 |  |
| Proboscidea | 3 | 60 |  |
| Tubulidentata | 1 | 60 |  |
| Macroscelidea | 15 | 60 |  |
| Tenrecidae | 33 | 60 |  |
| Chrysochloridae | 21 | 60 |  |
| Bradypodidae | 4 | 60 |  |
| Megalonychidae | 2 | 60 |  |
| Cyclopedidae | 1 | 60 |  |
| Myrmecophagidae | 3 | 60 |  |
| Dasypodidae | 21 | 60 |  |
| Pholidota | 8 | 60 |  |
| Mephitidae | 12 | 60 |  |
| Mustelidae | 59 | 60 |  |
| Procyonidae | 14 | 60 |  |
| Ailuridae | 1 | 60 |  |
| Otariidae | 16 | 60 |  |
| Odobenidae | 1 | 60 |  |
| Phocidae | 19 | 60 |  |
| Ursidae | 8 | 60 |  |
| Canidae | 35 | 60 |  |
| Felidae | 40 | 60 |  |
| Prionodontidae | 2 | 60 |  |
| Hyaenidae | 4 | 60 |  |
| Herpestidae | 33 | 60 |  |
| Eupleridae | 8 | 60 |  |
| Viverridae | 34 | 60 |  |
| Nandiniidae | 1 | 60 |  |
| Rhinocerotidae | 5 | 60 |  |
| Tapiridae | 4 | 60 |  |
| Equidae | 8 | 60 |  |
| Pteropodidae | 186 | 60 |  |
| Rhinolophidae | 77 | 60 |  |
| Megadermatidae | 5 | 60 |  |
| Craseonycteridae | 1 | 60 |  |
| Rhinopomatidae | 4 | 60 |  |
| Nycteridae | 16 | 60 |  |
| Emballonuridae | 51 | 60 |  |
| Phyllostomidae | 160 | 60 |  |
| Mormoopidae | 10 | 60 |  |
| Noctilionidae | 2 | 60 |  |
| Furipteridae | 2 | 60 |  |
| Thyropteridae | 3 | 60 |  |
| Mystacinidae | 2 | 60 |  |
| Myzopodidae | 1 | 60 |  |
| Vespertilionidae | 391 | 60 |  |
| Miniopteridae | 19 | 60 |  |
| Molossidae | 100 | 60 |  |
| Natalidae | 8 | 60 |  |
| Monodontidae | 2 | 60 |  |
| Phocoenidae | 6 | 60 |  |
| Delphinidae | 34 | 60 |  |
| Iniidae | 1 | 60 |  |
| Pontoporiidae | 1 | 60 |  |
| Lipotidae | 1 | 60 |  |
| Hyperoodontidae | 21 | 60 |  |
| Platanistidae | 2 | 60 |  |
| Kogiidae | 2 | 60 |  |
| Physeteridae | 1 | 60 |  |
| Balaenopteridae | 7 | 60 |  |
| Eschrichtiidae | 1 | 60 |  |
| Neobalaenidae | 1 | 60 |  |
| Balaenidae | 4 | 60 |  |
| Hippopotamidae | 2 | 60 |  |
| Bovidae | 143 | 60 |  |
| Moschidae | 7 | 60 |  |
| Cervidae | 51 | 60 |  |
| Giraffidae | 2 | 60 |  |
| Antilocapridae | 1 | 60 |  |
| Tragulidae | 8 | 60 |  |
| Suidae | 19 | 60 |  |
| Tayassuidae | 3 | 60 |  |
| Camelidae | 4 | 60 |  |
| Soricidae | 376 | 60 |  |
| Erinacidae | 24 | 60 |  |
| Talpidae | 39 | 60 |  |
| Solenodontidae | 4 | 60 |  |
| Aplodontidae | 1 | 60 |  |
| Sciuridae | 278 | 60 |  |
| Gliridae | 28 | 60 |  |
| Heteromyidae | 60 | 60 |  |
| Geomyidae | 40 | 60 |  |
| Castoridae | 2 | 60 |  |
| Muridae | 730 | 60 |  |
| Cricetidae | 681 | 60 |  |
| Nesomyidae | 61 | 60 |  |
| Spalacidae | 36 | 60 |  |
| Dipodidae | 51 | 60 |  |
| Peditidae | 2 | 60 |  |
| Anomaluridae | 7 | 60 |  |
| Ctenodactylidae | 5 | 60 |  |
| Diatomyidae | 1 | 60 |  |
| Hystricidae | 11 | 60 |  |
| Thryonomyidae | 2 | 60 |  |
| Petromuridae | 1 | 60 |  |
| Bathyergidae | 16 | 60 |  |
| Myocastoridae | 1 | 60 |  |
| Echimyidae | 90 | 60 |  |
| Capromyidae | 20 | 60 |  |
| Ctenomyidae | 66 | 60 |  |
| Octodontidae | 13 | 60 |  |
| _Abrocomidae | 10 | 60 |  |
| Chinchillidae | 7 | 60 |  |
| Dinomyidae | 1 | 60 |  |
| Erethizontidae | 16 | 60 |  |
| Caviidae | 18 | 60 |  |
| Agoutidae | 2 | 60 |  |
| Dasyproctidae | 13 | 60 |  |
| Ochotonidae | 30 | 60 |  |
| Leporidae | 60 | 60 |  |
| Scandentia | 20 | 60 |  |
| Dermoptera | 2 | 60 |  |
| Hominidae | 7 | 60 |  |
| Hylobatidae | 14 | 60 |  |
| Cercopithecidae | 81 | 60 |  |
| Cebidae | 56 | 60 |  |
| Pitheciidae | 40 | 60 |  |
| Atelidae | 24 | 60 |  |
| Tarsiidae | 7 | 60 |  |
| Loridae | 9 | 60 |  |
| Galagonidae | 19 | 60 |  |
| Daubentoniidae | 1 | 60 |  |
| Indridae | 11 | 60 |  |
| Megalopidae | 2 | 60 |  |
| Lemuridae | 19 | 60 |  |
| Cheriogaleidae | 32 | 60 |  |

**Supporting Table S2 References**

1. M.D. Guiry, & G.M. Guiry, *AlgaeBase.* (World-wide electronic publication, National University of Ireland, Galway. http://www.algaebase.org; Accessed September 2009).

2. H. S. Yoon, *et al*., D. *BMC Evol. Biol.* 6 78 (2006).

3. P. J. Keeling, *Am. J. Bot.* 91, 1481 (2004).

4. C. Van Den Hoek, D. G. Mann, D. G., H. M. Jahns, *Alage: An Introduction to Phycology.* (The Cambrige University Press, Cambridge UK 1995).

5. S. Magallon, K. W. Hilu, in *The Timetree of Life*, S. B. Hedges, S. Kumar, Eds. (Oxford University Press, UK 2009). Pp. 133-137

6. M. R. Crosby, R. E. Magill, B. Allen, S. He, *A Checklist of the Mosses* (Missouri Botanical Garden, St. Louis 1999).

7. L. Söderström, A. Hagborg, M. von Konrat, M. Renner, L. Gog, J. Engel. *Early Land Plants Today* (http://www.early-land-plants-today.org/ Accessed September 2009).

8. Smith, A. R., Pryer, K. M., Schuettpelz E., Korall, P., Schneider, H., & Wolf, P. G. *Taxon* 55 705-731 (2006).

9. M. Hassler, B. Swale, *Checklist of world Ferns* (http://homepages.caverock.net.nz/~bj/fern/ Accessed September 2009).

10. S. Renner. in *The Timetree of Life*, S. B. Hedges, S. Kumar, Eds. (Oxford University Press 2009). Pp. 157–160.

11. S. -M. Chaw, T. W. Walters, C.-C. Chang, S.-H. Hu, S.-H. Chen, *Mol. Phylogenet. Evol.* 37, 214 (2005).

12. P. F. Stevens,*Angiosperm Phylogeny Website. Version 9* (http://www.mobot.org/MOBOT/research/APweb/. 2009).

13. L. Watson, M.J. Dallwitz, *The families of flowering plants: descriptions, illustrations, identification, and information retrieval.* (http://delta-intkey.com (Accessed September 2009).

14. The Royal Botanic Gardens and Domain Trust. *PlantNET - The Plant Information Network System of The Royal Botanic Gardens and Domain Trust, Sydney, Australia*, (http://plantnet.rbgsyd.nsw.gov.au. Accessed September 2009).

15. M. P. Kirk, P. F. Cannon, D. W. Minter, J. A. Staplers, *Dictionary of the Fungi* *10^th^ Edition*. (CABI publishing Oxford, U.K. 2008).

16. N. King et al., *Nature* 451, 783 (2008).

17. M. Dohrmann, D. Janussen, J. Reitner, A. G. Collins, G. Wörheide, *Sys. Biol.* 57, 388 (2008).

18. J. N. A. Hooper, R. W. M van Soest, (Eds) *Systema Porifera: a guide to the classification of sponges*. (Kluwer Academic, New York 2002).

19. Mills, C.E. Phylum *Ctenophora: list of all valid species names*. (http://faculty.washington.edu/cemills/Ctenolist.html. Accessed October 2009)*.*

20. A. D. Rogers in *The Timetree of Life*, S. B. Hedges and S. Kumar, Eds. (Oxford University Press, UK 2009). Pp. 233–238

21. P. Schuchert, *The Hydrozoa Directory.* (http://www.ville-ge.ch/mhng/hydrozoa/hydrozoa-directory.htm Accessed October 2009).

22. P. Myers, 2002. *Platyhelminthes, Animal Diversity Web*. (http://animaldiversity.ummz.umich.edu/site/accounts/information/Platyhelminthes.html. Accessed December 2009).

23. R. Gibson, *J. Nat. Hist.* 29, 27 (1995).

24. R. E. Young, M. Vecchione, K. M. Mangold *Tree of Life: Index to Cephalopod Taxa* (http://tolweb.org/accessory/Index_to_Cephalopod_Taxa?acc_id=585 Accessed December 2009)*.*

25. G. Steiner, & A. R. Kabat, *Zoosystema* 23, 433 (2001).

26. G.W. Rouse, *Annelida (Segmented Worms)*. *Encyclopedia of Life Sciences*. (John Wiley & Sons, Ltd. 2002)

27. M. Blaxter, in *The Timetree of Life*, S. B. Hedges and S. Kumar, Eds. (Oxford University Press UK 2009). Pp. 247–250

28. D. Huang, J. Vannier, J.-Y. Chen, *Geobios* 37, 217 (2004).

29. J. Adis, M. S. Harvey, *Stud. on Neotrop. Fauna E.* 35, 139(2000).

30. X. Xia, *Sys. Biol.* 49, 87 (2000).

31. N. I. Platnick, *The world spider catalog, version 10*. (American Museum of Natural History http://research.amnh.org/iz/spiders/catalog. DOI: 10.5531/db.iz.0001. 2009).

32. C. P. Arango, W. C. Wheeler, *Cladistics* 23, 255 (2007).

33. R. C. Brusca, G. J. Brusca, *Invertebrates* *2^nd^ Edt.*  (Sinauer Associates 2003).

34. S. De Grave et al. *Raffles B. Zool.* 21, 1 (2009).

35. *GBIF Data Portal* (data.gbif.org Accessed December 2009).

36. W. E. Newman, *Zoosystema* 22, 71- 84 (2000).

37. M. Pérez-Losada, G. Bond-Buckup, C. G. Jara, K. A. Crandall, *Sys. Biol.* 53, 767(2004).

38. M. Shimomura, T. Akiyama, *J. Crustacean Biol.* 28, 572 (2008).

39. S. Koenemann, M. Hoenemann, T. Stemme (Eds) *World Remipedia Database*. (http://www.marinespecies.org/remipedia. Accessed December 2009).

40. J. Hallan, *The Biology Catalogue* (http://insects.tamu.edu/research/collection/hallan/ Accessed December 2009).

41. N.P. Kristensen, (Ed.) *Lepidoptera: Moths and butterflies Vol* *1: Evolution, Systematics and Biogeography*. de Gruyter Inc (1999).

42. P. C. Barnard et al. *The Trichoptera World Checklist*. (Available online at http://www.clemson.edu/cafls/departments/esps/database/trichopt/ Accessed December 2009).

43. T. R. New, G. Theischinger. *Megaloptera (Alderflies, Dobsonflies)*. de Gruyter (1993).

44. H. Aspöck, *Acta Zool. Hung.* 48 (2002).

45. J. D. Oswald, *Neuropterida Species of the World. Version 2.0*. (http://lacewing.tamu.edu/Species-Catalogue/. Accessed on December 2009).

46. *Hymenoptera Online Database* (http://hol.osu.edu/ Accessed August 2009)

47. S.J. Brands, *The Taxonomicon. Universal Taxonomic Services*, (Zwaag, The Netherlands. http://taxonomicon.taxonomy.nl/ Accessed September 2009).

48. *A comprehensive list of extant hemichordate species*. (https://www.webdepot.umontreal.ca/Usagers/cameroc/MonDepotPublic/Cameron/Species.html. Accessed December 2009)

49. R. Froese, D. Pauly, (Eds) *FishBase*. (Online at http://www.fishbase.org/ Accessed December 2009).

50. *AmphibiaWeb: Information on amphibian biology and conservation* (Berkeley, California: <http://amphibiaweb.org/>. Accessed June 2009).

51. P. Uetz, et al., *The Reptile Database* (http://www.reptile-database.org, Accessed June 2009).

52. A. J. Baker, & S. L. Pereira in *The Timetree of Life*, S. B. Hedges and S. Kumar, Eds. (Oxford University Press, U.K. 2009). Pp. 412–414

53. S. L. Pereira, A. J. Baker, in *The Timetree of Life*, S. B. Hedges and S. Kumar, Eds. (Oxford University Press, U.K. 2009). Pp. 415–418.

54*. Birdlife International* (http://www.birdlife.org/datazone/species/search Accessed June 2009).

55. M. Brooke, *Albatrosses and Petrels Across the World*. (Oxford University Press, U.K., 2004).

56. J.W. Brown, D.P. Mindell, in The Timetree of Life. Eds. S.B. Hedges and S. Kumar. (Oxford University Press, U.K. 2009). Pp 454 - 456

57. J.W. Brown, D.P. Mindell, in *The Timetree of Life*, S. B. Hedges and S. Kumar, Eds. (Oxford University Press, U.K. 2009). Pp. 436–439

58. W.S. Moore, K.J. Miglia in *The Timetree of Life*. S.B. Hedges and S. Kumar, Eds. (Oxford University Press, U.K. 2009). Pp 445-50.

59. J.W. Brown, D.P. Mindell, in *The Timetree of Life*, S. B. Hedges and S. Kumar, Eds. (Oxford University Press, U.K. 2009). Pp. 451–453.

60. D. E. Wilson, D. M. Reeder, (Eds). *Mammal Species of the World. A Taxonomic and Geographic Reference 3^rd^ ed* (Johns Hopkins University Press 2005)
